# Supplementary material for: The rotamer of the second-sphere histidine in AA9 lytic polysaccharide monooxygenase is pH dependent
Source: Biophys J. 2024 Apr 2;123(9):1139–51. doi: 10.1016/j.bpj.2024.04.002 (PMC11079946; doi:10.1016/j.bpj.2024.04.002)
Supplement: Document S1. Figures S1–S10, Tables S1, and S2 [file mmc1.pdf]

Biophysical Journal, Volume *123*

Supplemental information

The rotamer of the second-sphere histidine in AA9 lytic polysaccharide monooxygenase is pH dependent

Ingvild Isaksen, Suvamay Jana, Christina M. Payne, Bastien Bissaro, and Åsmund K. Røhr

This file includes:

1. List of Supplementary Figures and Tables
2. Supplementary Figures S1 to S10 and Supplementary Tables 1 to 2
3. Example ORCA input file (stacking His in HIE state and Cu(II))

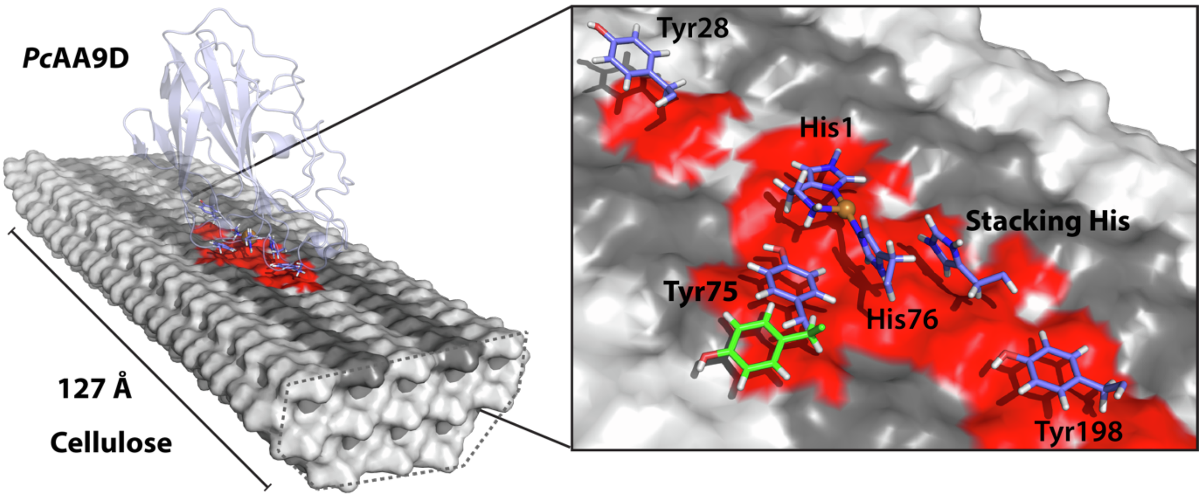

Fig. S1. Constant pH simulations of *Pc*AA9D on cellulose. The direction of cellulose chains forming the crystal is indicated by two chains with dark shades on the surface. The surface interacting with Tyr residues and the active site is colored red. Tyr28 and Tyr198 align along a cellulose chain. The alternative Tyr75 rotamer is indicated in green. The copper atom is shown as orange sphere. When bound to the substrate, the stacking His does not form any hydrogen bonds to the cellulose surface, and its flexibility is sterically restricted by the association of the enzyme with cellulose.

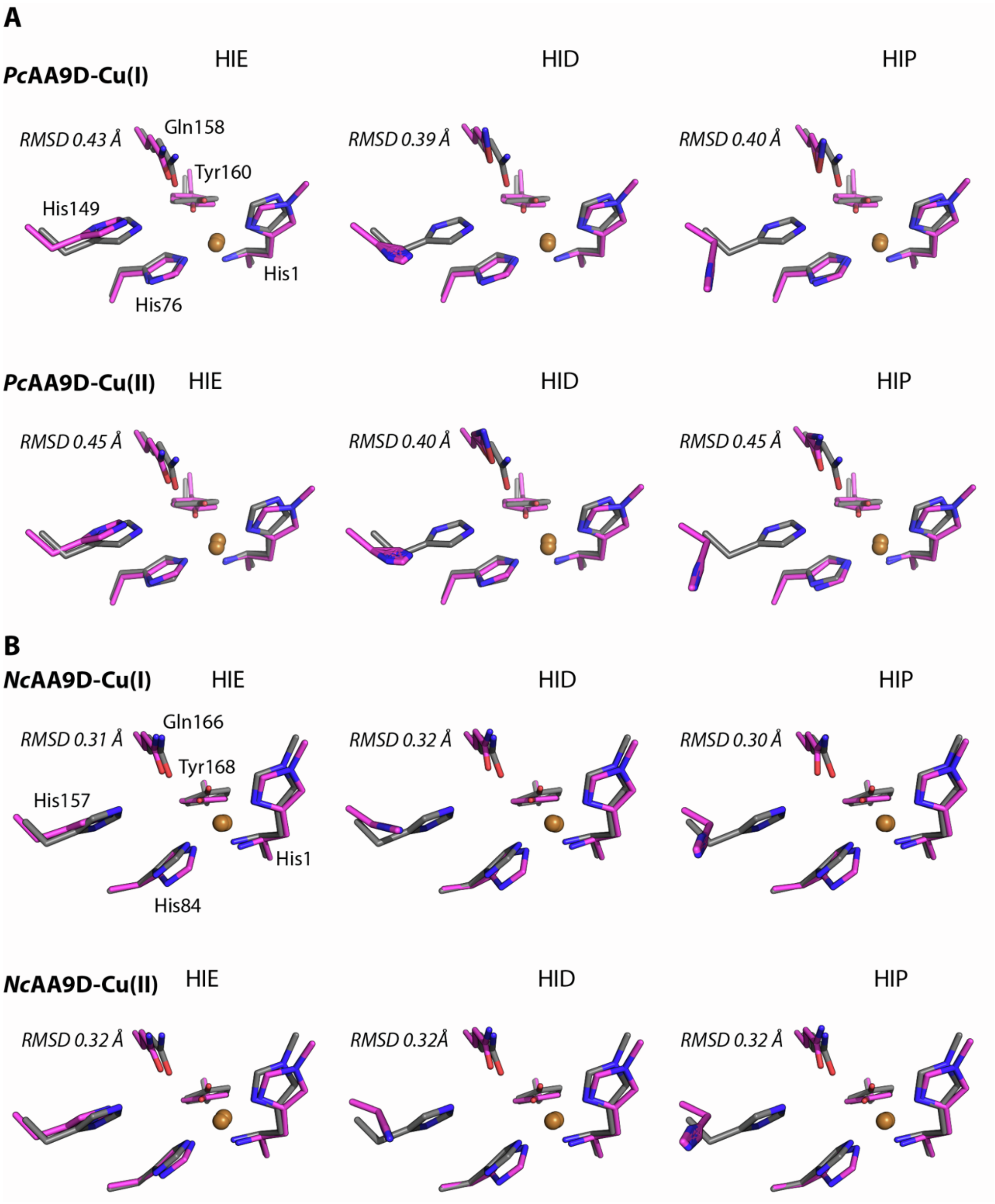

Fig. S2. Comparing averaged structures of *Pc*AA9D and *Nc*AA9D to respective crystal structures. Here, we assess the quality of the AA9 force field parameters developed in this study and evaluate how different protonation states of the stacking His behave during MD simulations in aqueous environments in the absence of substrate. The respective crystal structures, PDBid 4B5Q for *Pc*AA9D and PDBid 4EIR for *Nc*AA9D, are shown with grey carbon, while the averaged MD models from 100 ns of simulation are shown with magenta carbon atoms. The data for *Pc*AA9D are presented in panel A and the *Nc*AA9D data in panel B. The side chain of the HID or HIP state of the stacking His is typically disordered, and this is reflected by the non-physical averaged positions of the atoms in these models. Note that the side chain of the HIP state always has a conformation pointing away from the active site pocket. The RMSD values are calculated using the "super" function in PyMOL and include all the atoms of the displayed amino acids except those of the stacking His.

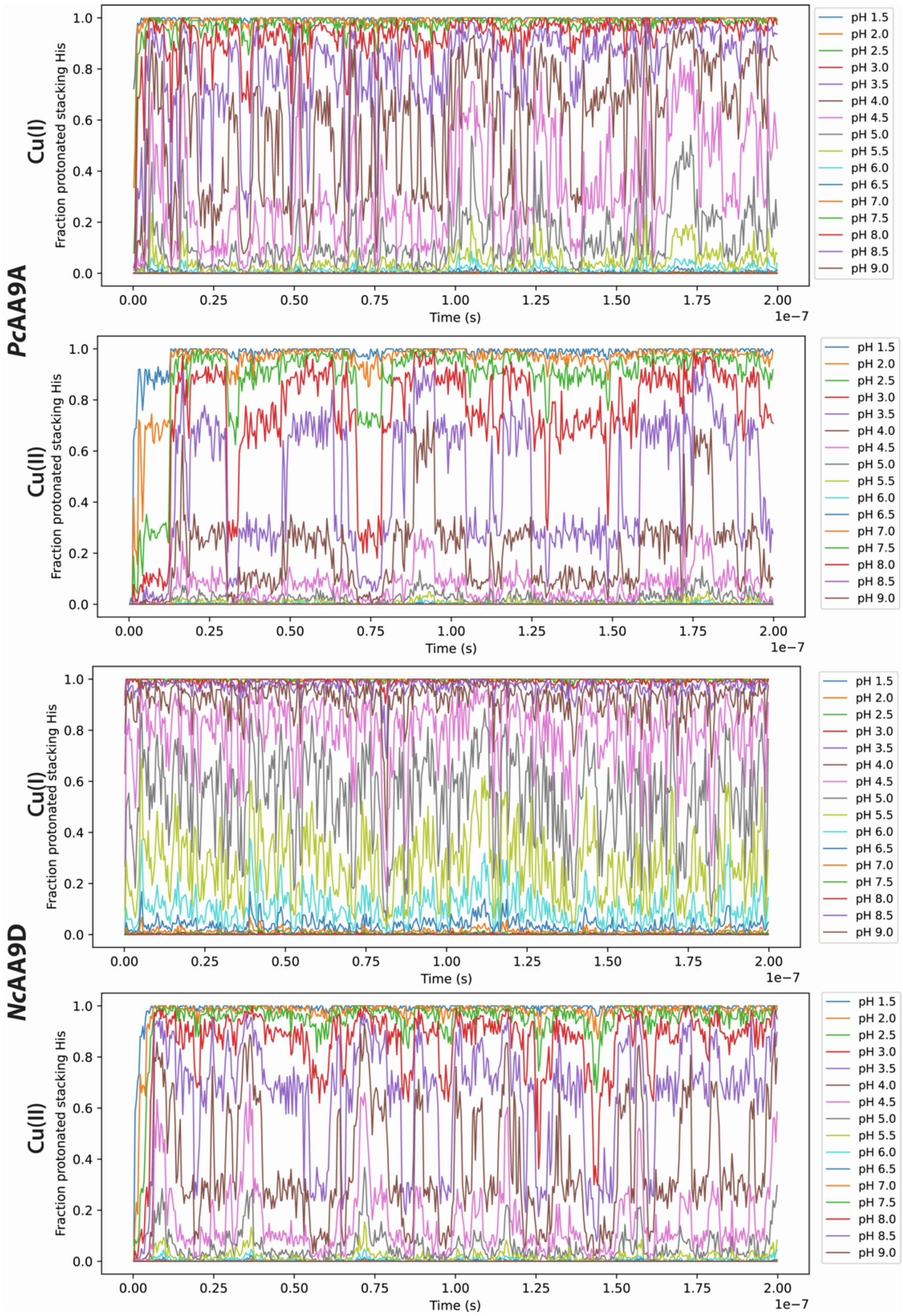

Fig. S3. Fraction of the HIP protonation state throughout the constant pH simulations. The panels show how the fraction of the HIP state changes over time at different pH-values. The data were prepared by sorting the replica exchange cpout files according to pH by the program cphstats (AmberTools23) and then the fractions of HIP were calculated in chunks of 200 000 steps. The simulation time for each pH-replica was 200 ns.

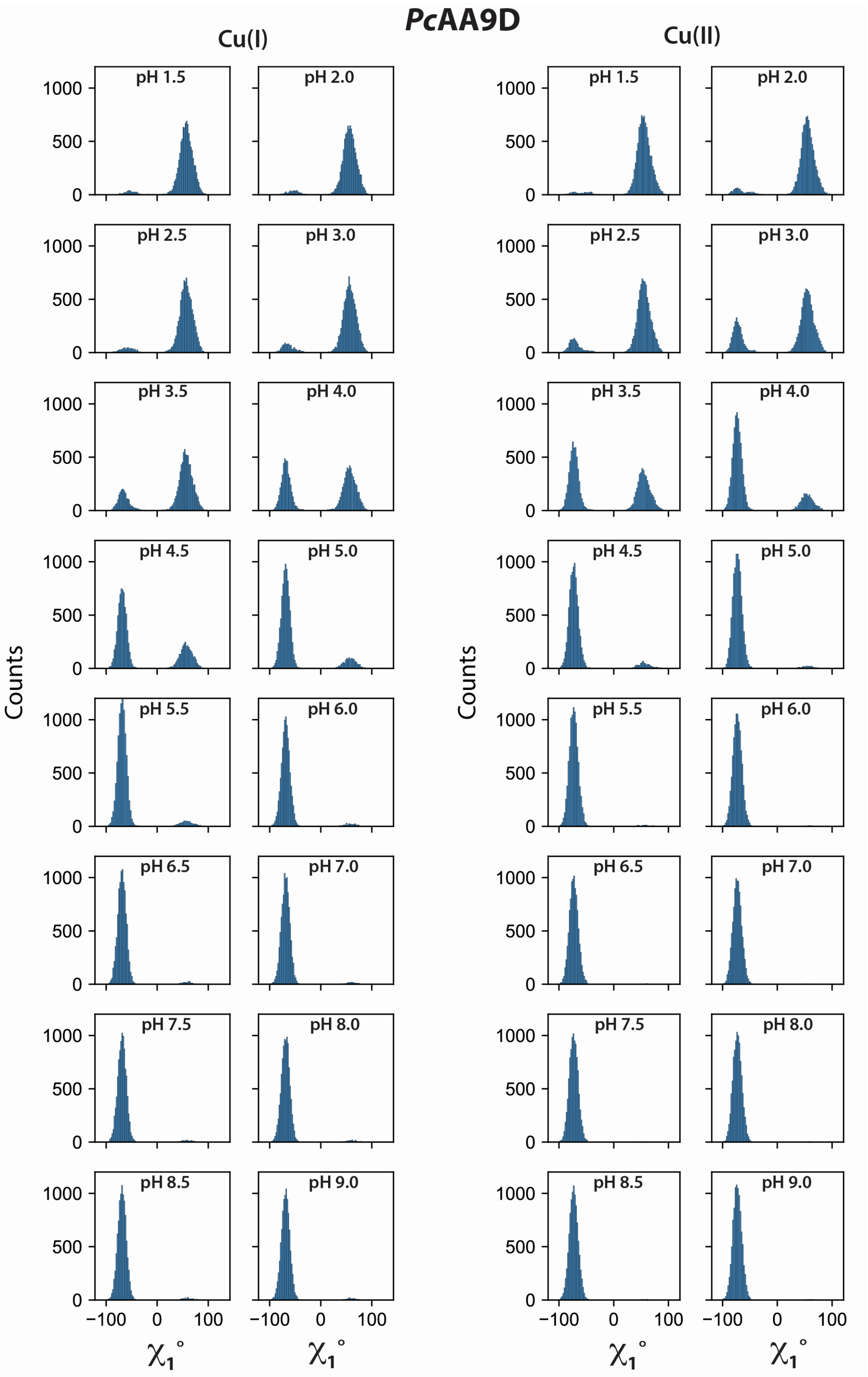

Fig. S4. Each panel show the populations of the stacking His rotamers different pH values in constant pH simulations for *Pc*AA9D. The panels show that two populations of the stacking His rotamers are observed, the "inward" (χ_1 ~ -70°) and "outward" (χ_1 ~ 60°) conformations, at different pH-values. The data were prepared by sorting the replica exchange trajectories according to pH by the program ccptraj (AmberTools23). The simulation time for each pH-replica was 200 ns.

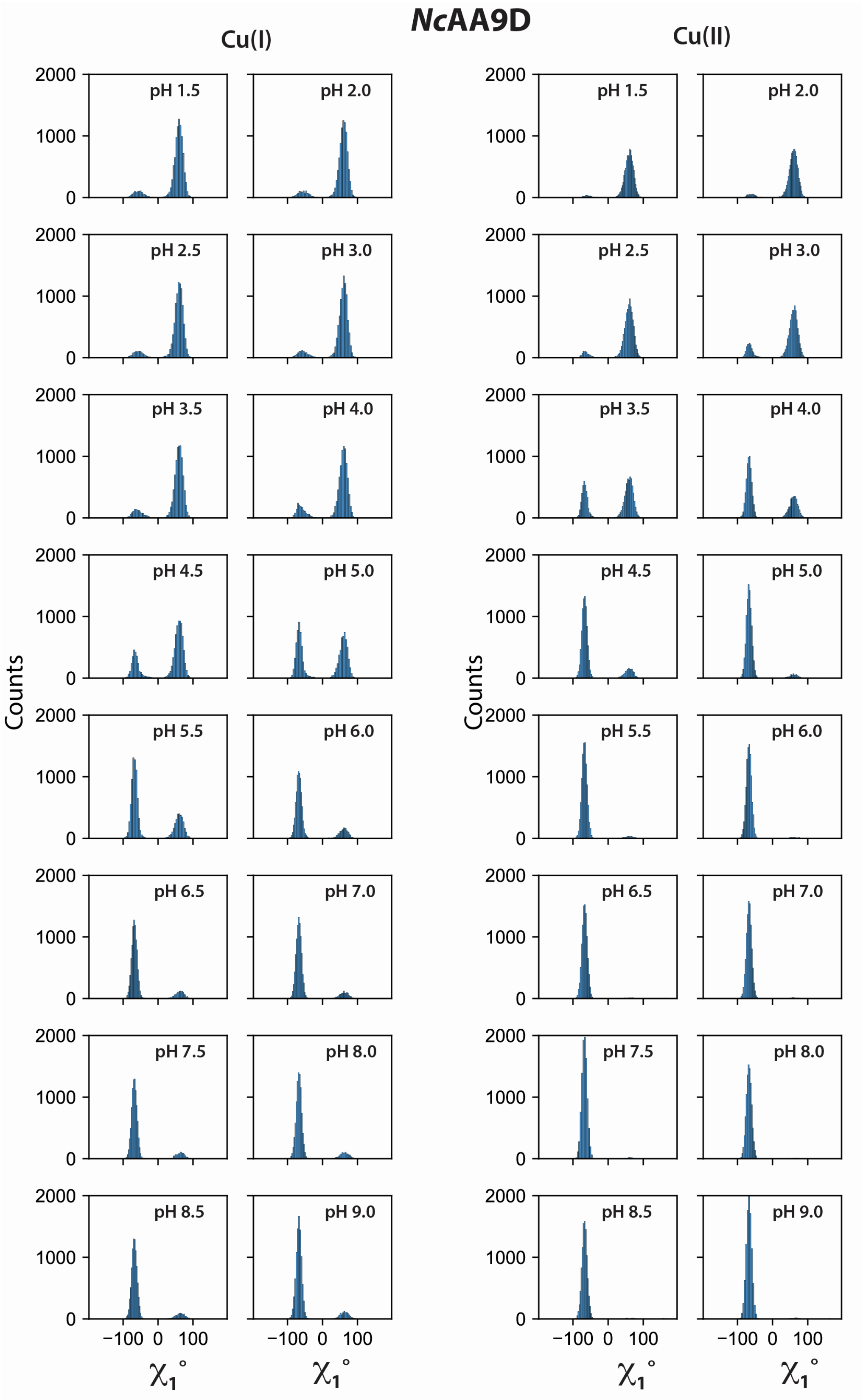

Fig. S5. Each panel show the populations of the stacking His rotamers different pH values in constant pH simulations for *Nc*AA9D. The panels show that two populations of the stacking His rotamers are observed, the "inward" (χ_1 ~ -70°) and "outward" (χ_1 ~ 60°) conformations, at different pH-values. The data were prepared by sorting the replica exchange trajectories according to pH by the program ccptraj (AmberTools23). The simulation time for each pH-replica was 200 ns.

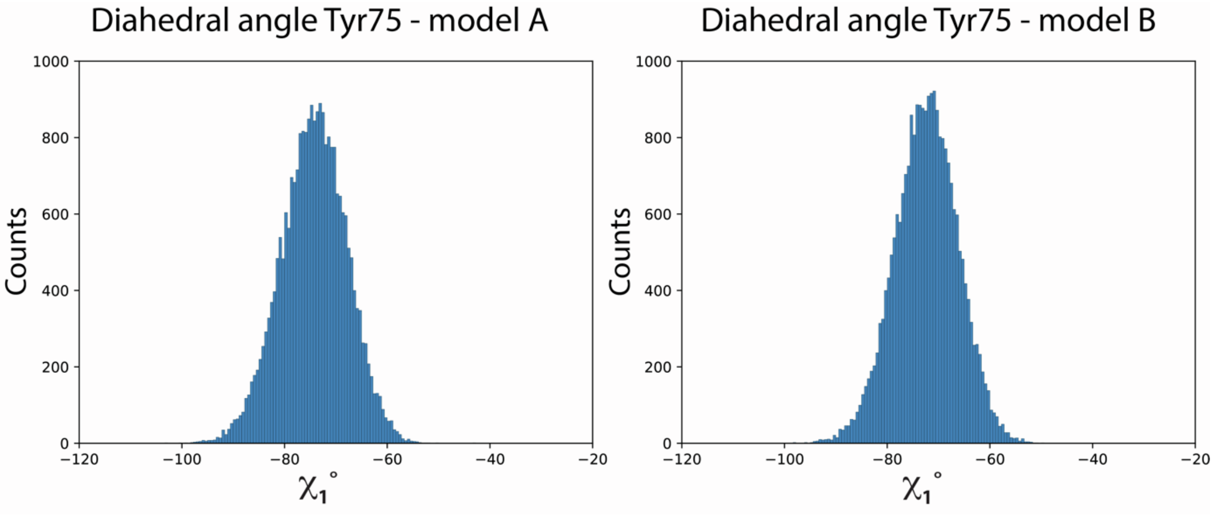

Fig. S6. Monitoring the Tyr75 χ_1 -rotamer in simulations of *Pc*AA9D on cellulose. The figures show how the Tyr160 side chain in the two models A and B, that had the starting dihedrals χ_1 = -64.7° and χ_1 = 160.5°, respectively, display a single population. The distribution of χ_1 falls around -75° for both models in the 500 ns simulations which is the rotamer pointing towards the copper.

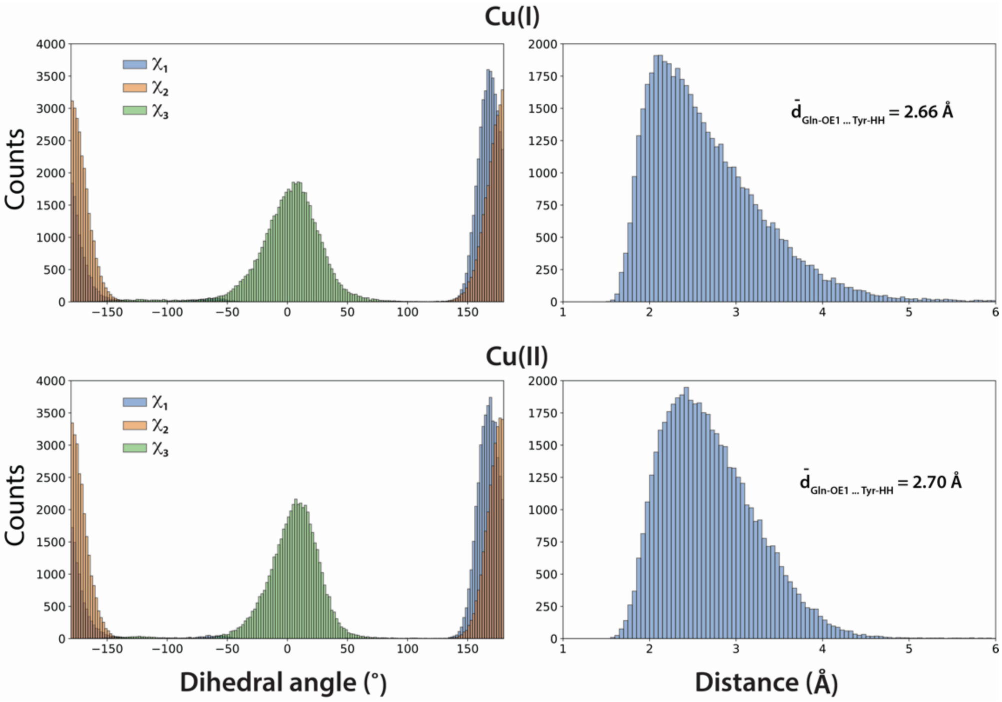

Fig. S7. Monitoring the Gln158 side chain rotamer and distance of Tyr160 interaction in MD simulations of *Pc*AA9D. The figure shows side chain dihedrals χ_1 to χ_3 (standard notation) that were monitored through 100 ns simulations of *Pc*AA9D in the Cu(I) and Cu(II) states. The distributions center around the values measured in the crystal structure (PDBid 4B5A chain A) that are χ_1 = 173.9°, χ_2 = 174.4° and χ_3 = 2.9°. The distance between the

hydrogen of the Tyr160 hydroxyl group and the Gln158 side chain carbonyl has a maximum around 2.3 Å, indicating frequent formation of a hydrogen bond.

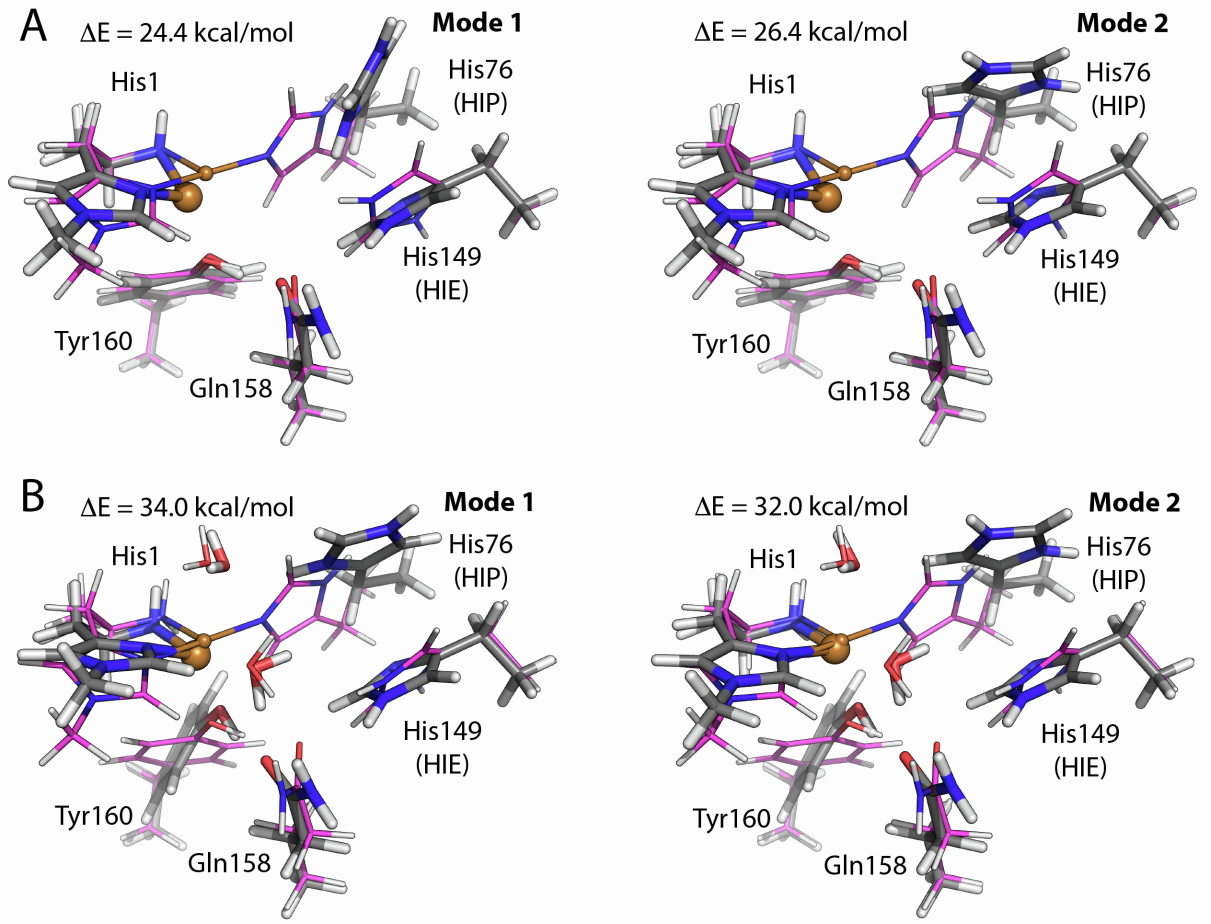

Fig. S8. Geometry optimized active site models of *Pc*AA9D with displaced His76. The DFT geometry optimized models where the models with displaced His76 are compared to the copper coordinating model (with the stacking His in the HIE state) in the Cu(I) state is shown in panel (A). The same comparison with Cu(II) is provided in panel (B). The difference in energy between the copper binding (with the stacking His in the HIP state) and displaced models are indicated, and for all models the copper coordinating version is predicted to be most stable.

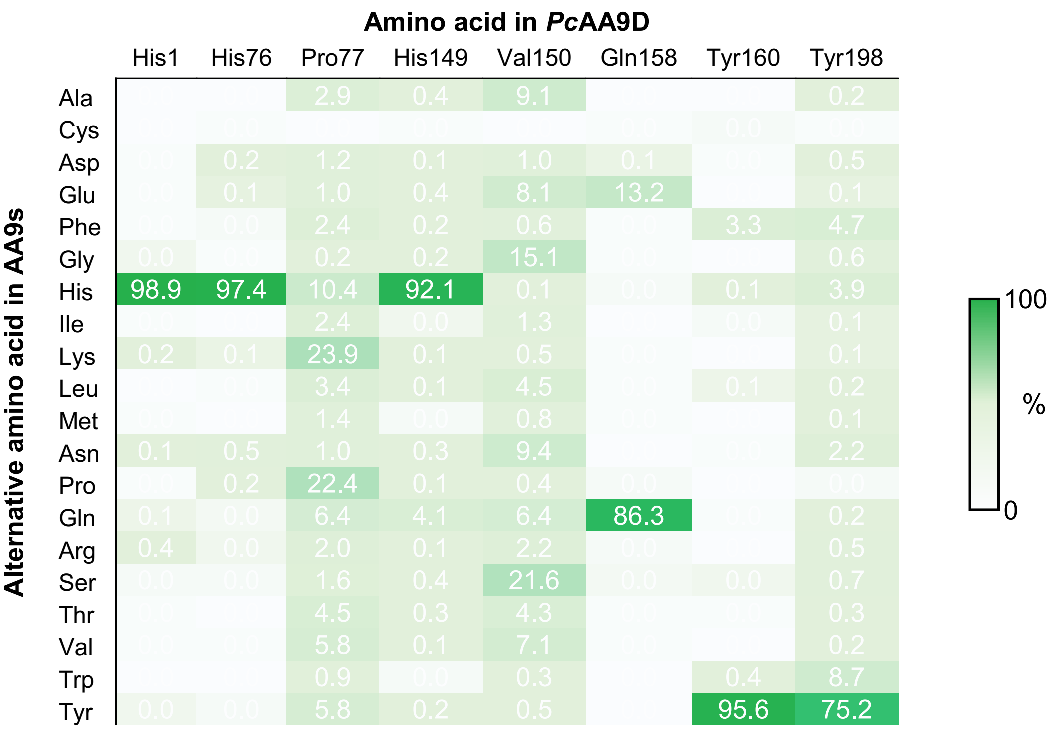

Fig. S9. Heatmap of the conservation score of key active site residues in AA9 LPMOs. The figure shows the conservation score (expressed as percentage), calculated on the basis of a multiple sequence alignment using 16,248 AA9 sequences, for positions equivalent to the copper-coordinating histidines (His1 and His76, *Pc*AA9D numbering), the stacking histidine (His149), catalytic helpers (Gln158 and Tyr160), and other second sphere residues highlighted in the present manuscript (Pro77, Val150 and Tyr198).

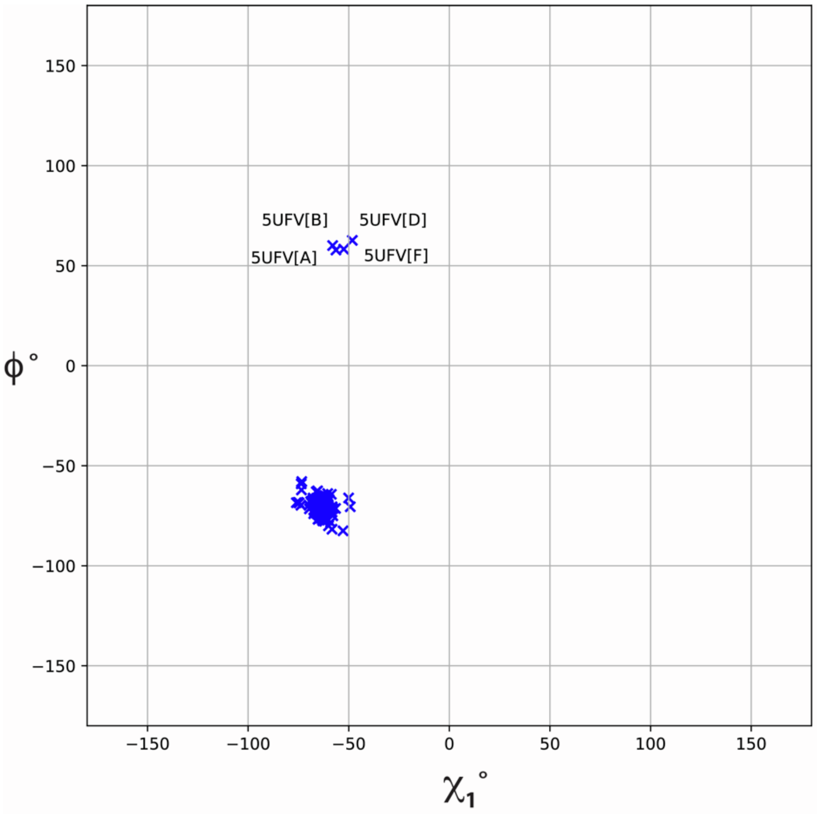

Fig. S10. Dihedral angles of the stacking His in AA9 LPMOs. The figure shows dihedral angles of the backbone N-C_α (ϕ) and the C_α - C_β (χ_1) (see **Fig. 1** for details). There are 89 structures included, some of them have multiple chains. There is a tight correlation between the ϕ and χ_1 values, indicating the a steric clash (see **Fig. 5**) will occur between the proton on the N_δ of the stacking His and the same residue amide hydrogen in most of the structures (the exception is from PDBid 5UFV that display the "outward" conformation in four of the monomers in the asymmetric unit). The included 89 PDB structures are 5NLT, 6YDC, 6YDD, 6YDE, 6YDF, 6H1Z, 6HA5, 6HAQ, 7EXK, 5NNS, 5ACF, 5ACG, 5ACH, 5ACI, 5ACJ, 5N04, 5N05, 5NKW, 5NLN, 5NLO, 5NLP, 5NLQ, 5NLR, 5NLS, 6YDG, 7NIM, 7NIN, 7PQR, 7PXI, 7PXJ, 7PXK, 7PXL, 7PXM, 7PXN, 7PXR, 7PXS, 7PXT, 7PXU, 7PXV, 7PXW, 7PYD, 7PYE, 7PYF, 7PYG, 7PYH, 7PYI, 7PYL, 7PYM, 7PYN, 7PYO, 7PYP, 7PYQ, 7PYU, 7PYW, 7PYX, 7PYY, 7PYZ, 7PZ0, 7NTL, 4EIR, 5TKF, 5TKG, 5TKH, 5TKI, 7T5C, 7T5D, 7T5E, 4QI8, 4EIS, 4D7U, 4D7V, 5FOH, 4B5Q, 7A8V, 2YET, 3ZUD, 7PZ3, 7PZ4, 7PZ5, 7PZ6, 7PZ7, 7PZ8, 7Q1K, 5UFV, 3EII, 3EJA, 2VTC, 5O2W and 5O2X.

Table S1. AMBER force field parameters for the AA9 LPMO active site with Cu(I) or Cu(II)

Bond stretching

	r_0 (Å)	k_r (kcal mol^{-1} Å^{-2})
NB-CU	1.991	83.1
N3-CU	2.090	39.2
OH-CU	2.653$^{Cu(I)}$, 2.371$^{Cu(II)}$	30.0
Extra parameters for methyl-group on His1		
CR-N*	1.376	431.6
CW-N*	1.376	431.6
CT-N*	1.456	334.7

Bond angle bending

	θ_0 (°)	k_θ (kcal mol^{-1} rad^{-2})
CU-N3-CX	117.1	84.4
CU-N3-H	107.0	46.5
N3-CU-NB	92.5	74.1
N3-CU-OH	92.6	37.6
NB-CU-OH	98.0	12.4
NB-CU-NB	161.8	42.2
CU-OH-C	131.1	36.4
CU-OH-HO	109.8	25.0
CU-NB-CR	127.1	77.8
CU-NB-CC	125.5	83.3
CU-NB-CV	125.5	83.3
Extra parameters for methyl-group on His1		
CC-CW-N*	121.6	67.5
N*-CR-NB	125.5	69.4
CR-N*-CW	104.3	70.5
CR-N*-CT	109.5	67.6
H5-CR-N*	123.7	49.2
H4-CW-N*	120.0	50.0
HC-CT-N*	109.4	49.8
CT-N*-CW	109.5	67.6

Charges Cu(I) AA9 active site

Cu(I) 0.1000

N-terminal His		Internal His		Buried Tyr	
Atom name	*Charge*	*Atom name*	*Charge*	*Atom name*	*Charge*
N	-0.4490	CB	-0.0819	CB	0.2619
H1	0.2268	HB2	0.0801	HB2	-0.0322
H2	0.2268	HB3	0.0801	HB3	-0.0322
CA	0.3760	CG	0.0130	CG	-0.1258
HA	0.0132	ND1	-0.1252	CD1	-0.1067
CB	-0.3619	HD1	0.3153	HD1	0.1349
HB2	0.1172	CE1	-0.1127	CE1	-0.2657
HB3	0.1172	HE1	0.1739	HE1	0.1199
CG	0.3015	NE2	-0.0931	CZ	0.3706
ND1	-0.2969	CD2	-0.1080	OH	-0.4024
CE1	-0.1360	HD2	0.1126	HH	0.2803
HE1	0.1879	CA	0.0188		
NEM	0.3217				
CD2	-0.4212				
HD2	0.2180				
CME	-0.3185				
HM1	0.1348				
HM2	0.1348				
HM3	0.1348				
C	0.6123				

Charges Cu(II) AA9 active site

Cu(II)	0.5843

N-terminal His		Internal His		Buried Tyr	
Atom name	*Charge*	*Atom name*	*Charge*	*Atom name*	*Charge*
N	-0.5957	CB	-0.1166	CB	0.2510
H1	0.3166	HB2	0.1063	HB2	-0.0183
H2	0.3166	HB3	0.1063	HB3	-0.0183
CA	0.2432	CG	0.1282	CG	-0.0978
HA	0.0765	ND1	-0.2043	CD1	-0.1255
CB	-0.1877	HD1	0.3551	HD1	0.1526
HB2	0.1129	CE1	-0.0776	CE1	-0.1884
HB3	0.1129	HE1	0.1947	HE1	0.1111
CG	0.0810	NE2	-0.1099	CZ	0.2680
ND1	-0.1266	CD2	-0.1978	OH	-0.4361
CE1	-0.2910	HD2	0.1114	HH	0.2875
HE1	0.2218	CA	0.25		
NEM	-0.1057				
CD2	0.1865				
HD2	0.2375				
CME	-0.2949				
HM1	0.1481				
HM2	0.1481				
HM3	0.1481				
C	0.6518				

Table S2. Analysis of populated states for His149 in cellulose-bound *Pc*AA9D in constant pH-replica exchange molecular dynamics simulations (starting model A and B)

Solvent pH	Fraction of protonated states recorded in 200 ns simulations for each pH					
	State 0 (HIP)		*State 1 (HID)*		*State 2 (HIE)*	
	**Model A**	**Model B**	**Model A**	**Model B**	**Model A**	**Model B**
1.50	0.000000	0.000002	0.004017	0.006266	0.995983	0.993732
2.00	0.000000	0.000001	0.004111	0.006476	0.995889	0.993523
2.50	0.000000	0.000000	0.004108	0.006498	0.995892	0.993502
3.00	0.000000	0.000000	0.004177	0.006443	0.995823	0.993557
3.50	0.000000	0.000000	0.004234	0.006401	0.995766	0.993599
4.00	0.000000	0.000000	0.004144	0.006296	0.995856	0.993704
4.50	0.000000	0.000000	0.004225	0.006193	0.995775	0.993807
5.00	0.000000	0.000000	0.004179	0.006039	0.995821	0.993961
5.50	0.000000	0.000000	0.004157	0.006173	0.995843	0.993827
6.00	0.000000	0.000000	0.004130	0.006208	0.995870	0.993792
6.50	0.000000	0.000000	0.004148	0.006185	0.995852	0.993815
7.00	0.000000	0.000000	0.004096	0.006282	0.995904	0.993718
7.50	0.000000	0.000000	0.004101	0.006236	0.995899	0.993764
8.00	0.000000	0.000000	0.004062	0.006245	0.995938	0.993755
8.50	0.000000	0.000000	0.004092	0.006187	0.995908	0.993813
9.00	0.000000	0.000000	0.004132	0.006191	0.995868	0.993809

Example ORCA input file:

```
# Orca input file
! UKS B3LYP OPT def2-SVP D4 TightSCF SlowConv

%base "b3lyp_opt"

%pal nprocs 10
    end

%basis
  newGTO Cu "def2-TZVPP" end
end

%geom
  ConnectFragments

  {1 2 C 15 23 }
  {1 3 C 15 38 }
  {1 4 C 15 53 }
  {1 5 C 15 68 }
  {1 6 O 15 87 }
  {1 7 O 15 88 }
  {1 8 O 15 91 }
  end
end

%scf
MaxIter 1500
DIISMaxEq 15
end

* xyz 2 2
N(1)   3.36069110761946    6.25010312328851   -15.35543576294581
H(1)   2.90695213375984    7.15845360886818   -15.52680163412305
H(1)   2.94976768176188    5.88514357866140   -14.48290882281649
C(1)   4.82849355357674    6.44022855073708   -15.15029603224286
H(1)   5.19747371120119    5.44630273419179   -14.82137099493967
C(1)   5.50741521182581    6.78457414659860   -16.47967652403444
H(1)   4.97419534099623    7.62376672611532   -16.97290914018203
H(1)   6.52564175887847    7.16267699992295   -16.26399994621672
C(1)   5.65000430013041    5.60273431070813   -17.41244343800138
N(1)   4.60491859329780    4.83026779422226   -17.77972134502170
C(1)   5.03453185690099    3.86145663412101   -18.61148475461291
H(1)   4.47435444009080    3.06202039279497   -19.09026594951698
N(1)   6.35801018189872    4.01523613272871   -18.77588060280792
```

C(1)	6.76670916585375	5.07994554815823	-18.05384558200822
H(1)	7.81142597506136	5.41108752452430	-18.04705962000941
C(1)	5.13742755943837	7.44157735162310	-14.06587436334866
C(1)	7.22370237407258	3.14590066017460	-19.59512346189016
H(1)	7.70546171456016	3.73800504341613	-20.39616073351435
H(1)	6.60715563207852	2.35106819004931	-20.05033836753547
H(1)	8.00300041461844	2.68594395600216	-18.95855362608271
H(1)	6.22696975932433	7.48046045340256	-13.86678321344335
H(1)	4.81449833151011	8.46769306692085	-14.34446776348778
H(1)	4.64035063753438	7.17253990549299	-13.11202913578676
C(2)	-3.09350422707110	5.23310231331244	-14.24881716939278
C(2)	-2.02911519606364	4.17167051820058	-14.49496454729120
H(2)	-1.52548180471928	3.89397808741075	-13.54764643633108
H(2)	-2.48674431181304	3.23152481892388	-14.88328856903453
C(2)	-0.95081193693749	4.57322893910912	-15.47317409418563
N(2)	-1.21992477688773	5.09578258990847	-16.69117941411969
H(2)	-2.15897190279197	5.24478994404343	-17.07135042139577
C(2)	-0.06343772013575	5.31305174786574	-17.34448011139885
H(2)	0.01417447601068	5.77224031990922	-18.33559612522308
N(2)	0.94762730585045	4.93855789374098	-16.54004250117959
C(2)	0.43491877511506	4.47123269224226	-15.38617893778095
H(2)	1.04535496393122	4.06374292712683	-14.57337208708744
H(2)	-3.83421043013390	4.86903076893024	-13.51080245371701
H(2)	-2.65897259452025	6.17362345189065	-13.85394096854520
H(2)	-3.66623885372595	5.48120609742847	-15.16754577947917
C(3)	-4.87822942918604	0.33248180216325	-18.03263060511988
C(3)	-4.36349689624197	1.75993321159828	-18.17094871265023
H(3)	-4.72866103437339	2.21833947770702	-19.11380524339295
H(3)	-4.77531327847072	2.38078072576520	-17.34447207025398
C(3)	-2.85545557324195	1.87711884689340	-18.12822809240209
N(3)	-2.13190376850070	1.51407306426481	-17.05226415570522
C(3)	-0.82821304784465	1.73836684717977	-17.28922255998767
H(3)	0.02162696114094	1.52769086268678	-16.63208401618738
N(3)	-0.71428585124539	2.26664963847666	-18.52444518560130
H(3)	0.15507300649415	2.40541290189572	-19.03900914015994
C(3)	-1.94713929192272	2.36757808605283	-19.06616367415684
H(3)	-2.09536360327059	2.73346248058655	-20.09007674541300
H(3)	-5.98539672495845	0.31281972865203	-18.00412886328978
H(3)	-4.54878785965205	-0.30170826880435	-18.88062457595024
H(3)	-4.50212273509173	-0.13070976997883	-17.09857702616906
C(4)	2.46342538909779	-2.24862361281552	-14.55483415153107
C(4)	2.43531365089472	-0.96005681587789	-15.36616351719054
H(4)	1.50057273229063	-0.40124586219339	-15.14582407920657
H(4)	3.25900650323302	-0.28333736340959	-15.05605543208095
C(4)	2.51598307042395	-1.18594418817485	-16.86857868823683
H(4)	3.48478675771435	-1.65559655674695	-17.15423103022532
H(4)	1.73444672192463	-1.90676435430981	-17.20088429479965

```
C(4)   2.35083347811388     0.09366153498599   -17.66523205342749
O(4)   2.22486716490969     1.18499829453458   -17.10305739913178
N(4)   2.35926421221942    -0.03434202270625   -18.99211750959856
H(4)   2.20157744164507     0.77081884795728   -19.59809683237365
H(4)   2.42141018006469    -0.94852703665452   -19.44412533749113
H(4)   2.40537736862526    -2.03727102630952   -13.46901328757420
H(4)   1.61069533255103    -2.91160022391984   -14.80995962434700
H(4)   3.39508724889639    -2.82666756925615   -14.72887624710202
C(5)   4.97723995173900     1.26048857221720    -9.48402082763512
C(5)   4.22328631232703     2.53090144084113    -9.86986930770160
H(5)   3.22138840661584     2.54421233232269    -9.39281469842577
H(5)   4.76481846306868     3.42474973098565    -9.49355837190387
C(5)   4.07428790616272     2.62128327160808   -11.37658215289970
C(5)   5.12229082333205     3.08658822698271   -12.16541305129597
H(5)   6.05825145652264     3.41489115357940   -11.68541441478491
C(5)   5.01002258852123     3.14509804719640   -13.54926797190256
H(5)   5.86601323369725     3.47953172816064   -14.16085320408370
C(5)   3.83975649485525     2.72039503920325   -14.15643320381172
O(5)   3.72647065078571     2.77485847197022   -15.53331738716652
H(5)    3.091854 2.135455 -15.884828
C(5)   2.78879873205647     2.24628340734727   -13.39566849630879
H(5)   1.88615269643895     1.87467668568720   -13.90289506449421
C(5)   2.91306794327773     2.18876125062277   -12.01178361543141
H(5)   2.07688319656763     1.79725463304458   -11.41140395926209
H(5)   5.99121299613359     1.24047652088591    -9.93362342786603
H(5)   5.09697279339550     1.17748332700502    -8.38491523963758
H(5)   4.44199387016382     0.35410796277802    -9.83411442025919
Cu(6)  2.83158546196694     4.99679449457430   -17.00300911912393
O(7)   2.63128933198794     3.92383420771523   -18.45571445751321
H(7)   1.76574533438357     4.07592516012055   -18.89735363986935
H(7)   2.49026671907397     2.99769504217166   -18.04671231529538
O(8)   2.87202350861426     6.71966816188550   -17.86825841557105
H(8)   2.20359498245672     7.43424300621560   -17.94524205831994
H(8)   3.44979415686138     6.84040209192311   -18.65130088032331
*

################################################################################
######
# JOB 2
################################################################################
######

$new_job

%base "b3lyp_sp"

! UKS B3LYP def2-TZVPP D4 TightSCF SlowConv
```

```
%pal nprocs 10
    end

%scf
MaxIter 1500
DIISMaxEq 15
end

* xyzfile 2 2
```
