# Supplementary material for: The rotamer of the second-sphere histidine in AA9 lytic polysaccharide monooxygenase is pH dependent
Source: Biophys J. 2024 Apr 2;123(9):1139–51. doi: 10.1016/j.bpj.2024.04.002 (PMC11079946; doi:10.1016/j.bpj.2024.04.002)
Supplement: Document S2. Article plus Supporting Material [file mmc2.pdf]

Biophysical Journal
Article

Biophysical Society

The rotamer of the second-sphere histidine in AA9 lytic polysaccharide monooxygenase is pH dependent

Ingvild Isaksen,[1] Suvamay Jana,[2] Christina M. Payne,[2] Bastien Bissaro,[1,3,*] and Åsmund K. Røhr[1,*]
[1]Faculty of Chemistry, Biotechnology and Food Science, Norwegian University of Life Sciences (NMBU), Ås, Norway; [2]Department of Chemical and Materials Engineering, University of Kentucky, Lexington, Kentucky; and [3]INRAE, Aix Marseille University, UMR1163 Biodiversité et Biotechnologie Fongiques, Marseille, France

ABSTRACT Lytic polysaccharide monooxygenases (LPMOs) catalyze a reaction that is crucial for the biological decomposition of various biopolymers and for the industrial conversion of plant biomass. Despite the importance of LPMOs, the exact molecular-level nature of the reaction mechanism is still debated today. Here, we investigated the pH-dependent conformation of a second-sphere histidine (His) that we call the stacking histidine, which is conserved in fungal AA9 LPMOs and is speculated to assist catalysis in several of the LPMO reaction pathways.Using constant-pH and accelerated molecular dynamics simulations, we monitored the dynamics of the stacking His in different protonation states for both the resting Cu(II) and active Cu(I) forms of two fungal LPMOs. Consistent with experimental crystallographic and neutron diffraction data, our calculations suggest that the side chain of the protonated and positively charged form is rotated out of the active site toward the solvent. Importantly, only one of the possible neutral states of histidine (HIE state) is observed in the stacking orientation at neutral pH or when bound to cellulose. Our data predict that, in solution, the stacking His may act as a stabilizer (via hydrogen bonding) of the Cu(II)-superoxo complex after the LPMO-Cu(I) has reacted with O_2 in solution, which, in fine, leads to H_2O_2 formation. Also, our data indicate that the HIE-stacking His is a poor acid/base catalyst when bound to the substrate and, in agreement with the literature, may play an important stabilizing role (via hydrogen bonding) during the peroxygenase catalysis.Our study reveals the pH titration midpoint values of the pH-dependent orientation of the stacking His should be considered when modeling and interpreting LPMO reactions, whether it be for classical LPMO kinetics or in industry-oriented enzymatic cocktails, and for understanding LPMO behavior in slightly acidic natural processes such as fungal wood decay.

SIGNIFICANCE Understanding how enzymes break down plant biomass is critical for both natural ecosystems and industrial processes such as biofuel production. This study focuses on a specific component of lytic polysaccharide monooxygenases (LPMOs)—a histidine residue—to unravel its possible role in the enzyme activity. Using advanced computational methods, we determined how this histidine behaves under different conditions and may contribute to the enzyme function. Our findings reveal that its orientation and function are pH dependent, offering new insights into how these enzymes work.

INTRODUCTION

Conversion of chitinous and lignocellulosic biomass into biofuels and other valuable commodities constitutes one of the major endeavors undertaken by scholars and industrials in the context of the emerging bioeconomy (1,2). A novel class of monocopper enzymes named lytic polysaccharide monooxygenases (LPMOs) was discovered to act as "decrystallizing" agents cleaving the polysaccharide chains of chitin and cellulose via an oxidative mechanism (3–6), thus disrupting the crystalline surface of these substrates (7–9). Glycoside hydrolases are thought to exploit the decrystallized polysaccharide chain ends displayed at such disrupted surfaces, resulting in overall boosted saccharification processes when they are combined with LPMOs. LPMOs are found in the three domains of life and are classified currently in eight families of the auxiliary activities (AAs) in the Carbohydrate Active Enzymes

Submitted October 9, 2023, and accepted for publication April 1, 2024.
*Correspondence: bastien.bissaro@inrae.fr or asmund.rohr@nmbu.no
Suvamay Jana's present address is Department of Chemical Engineering, Indian Institute of Technology Dharwad, Dharwad 580011, India
Editor: Chris Neale.
https://doi.org/10.1016/j.bpj.2024.04.002
© 2024 Biophysical Society.
This is an open access article under the CC BY license (http://creativecommons.org/licenses/by/4.0/).

(CAZy) database (10), namely AA9–11 and AA13–17. LPMOs are harnessed in various applications, such as in industrial biorefineries (11), and appear to be involved in an increasing range of biological processes (12).

The disruption action of LPMOs is known to entail a monocopper-catalyzed hydroxylation of the C1 and/or C4 of the glycosidic bond leading to bond cleavage (13). However, the exact LPMO catalytic mechanism is still not fully resolved (14). Since their discovery, it was thought that LPMOs used O_2 as co-substrate (5); however, in 2016, we showed that LPMOs could use H_2O_2 instead (15,16), and more efficiently than O_2 (17,18). Several independent studies, using various computational (19–21) and biochemical (17,22–25) approaches, validated that LPMOs do, indeed, use H_2O_2 as a co-substrate. In LPMO catalysis, two configurations should be distinguished: the substrate-bound LPMOs and in-solution (i.e., not bound) LPMOs (see recent review by Munzone et al. for an overview (14)). It is known and widely accepted that, in solution, reduced LPMOs (i.e., LPMO–Cu(I)) can facilitate the reduction of O_2 into H_2O_2 in the presence of a reductant (26), a reaction called the oxidase path (O path). In solution, LPMO–Cu(I) can also catalyze a peroxidase reaction (P path) (27) or undergo an irreversible inactivation reaction (I path) (16) in the presence of excessive amounts of H_2O_2. When bound to their substrate, LPMOs can use H_2O_2 as co-substrate in a peroxygenase reaction (R–H + $H_2O_2 \rightarrow$ R–OH + H_2O; PO path) (16,21). Whether or not O_2 can also be used directly, in a so-called monooxygenase reaction (R–H + O_2 + $2e^-$ + $2H^+ \rightarrow$ R–OH + H_2O; MO path), requiring the timely delivery of electrons and protons, remains an open question. However, no clear-cut experimental evidence for such a mechanism exists thus far. Knowledge of how the close environment of the catalytic center fine-tunes the oxidase (reduction of O_2 to H_2O_2) and peroxygenase activities of LPMOs is critical to understand these enzymes. Notably, by analogy with other O_2/H_2O_2-using enzymes, the occurrence of acid/base catalysis during O_2 and/or H_2O_2 activation, both in solution and in substrate-bound configuration, remains an open question.

Typically, LPMOs have an active site consisting of two conserved histidines, one being the N-terminal residue and both coordinating a single copper atom, defining a so-called histidine brace (28). Representative structures of AA9 and AA10 LPMO active sites are shown in Fig. 1 *A* and *B* (4,29).

Immediately below the surface-exposed active site, an aromatic side chain from either a tyrosine (Fig. 1 *A*) or a phenylalanine (Fig. 1 *B*) is found to pack against the copper ion. As part of the solvent-exposed active site, one or two polar or charged amino acid side chains, highly conserved within each LPMO family, point toward the copper ion from a distance of approximately 4–6 Å. In AA9s, these amino acids typically comprise a histidine/glutamine pair (Fig. 1 *A*), whereas, in AA10s, a single glutamate is usually the structural analog of the glutamine found in AA9s (Fig. 1 *B*). Site-directed mutagenesis experiments have shown that these second-coordination-sphere residues are important for catalysis (30–34). Recent computational studies have suggested that the second-sphere glutamate in the AA10 enzyme from the bacterium *Serratia marcescens* (*Sm*AA10A) has a role in regulating the access of small molecules such as O_2 and H_2O_2 to the enzyme active site (35), and a role in facilitating the reaction between H_2O_2 and the Cu(I) form of the LPMO during catalysis has also been suggested (21).

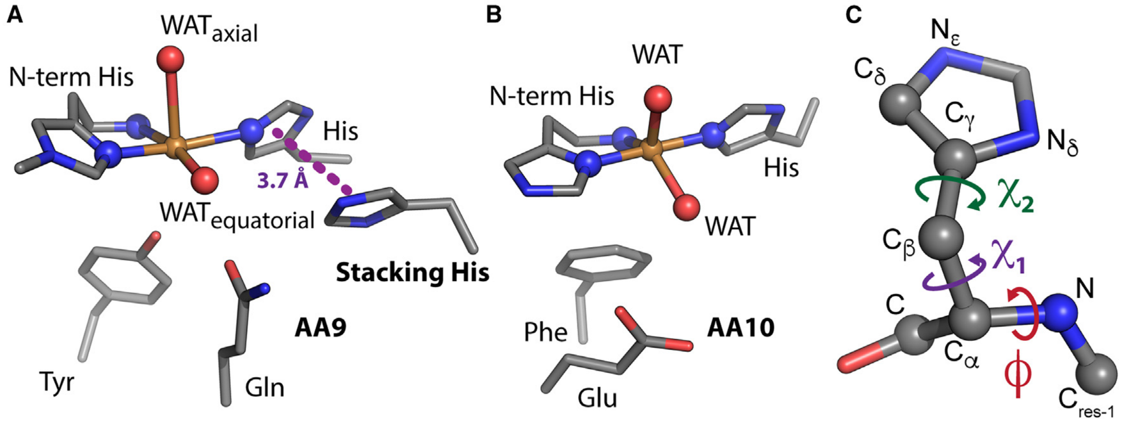

FIGURE 1 Structural comparison of AA9 and AA10 active sites. (*A*) Active site of the fungal, C1-oxidizing cellulose-active AA9A from *Thermoascus aurantiacus* (*Ta*AA9A, PDB: 2YET) (4), where the buried Tyr residue and the solvent-exposed Gln and stacking His are shown in addition to the histidine brace. (*B*) Active site of the bacterial chitin active AA10A from *Enterococcus faecalis* V583 (*Ef*AA10A, PDB: 4ALC) (29), where the buried Phe and solvent-exposed Glu close to the histidine brace are also displayed. The copper atom and the copper-coordinating atoms are shown as spheres. In the AA9, the two copper-coordinating water molecules (WAT) are found in axial and equatorial positions (assuming a distorted octahedral geometry), whereas the copper ligands displayed for the AA10 show a distorted trigonal bipyramidal geometry. (*C*) Illustration of the two nitrogen atoms in the imidazole ring of the histidine side chain that can be protonated (N_ε and N_δ), and the dihedral angles χ_1 (C-C_α-C_β-C_γ), χ_2 (C_α-C_β-C_γ-C_δ) and φ (C_{res-1}-N-C_α-C) that are measured for the stacking His in MD trajectories. To see this figure in color, go online.

The highly conserved histidine of the histidine/glutamine pair (Fig. 1 *A*) is found in close proximity to the histidine brace (see purple dotted line, 3.8 Å, in Fig. 1 *A*). We named this residue the stacking His because it forms a stacking interaction with the second Cu-coordinating histidine of the brace called the internal His. Histidine side chains (pK_a of 6.0 in water) can have three different protonation states, usually referred to as HID (N_δ protonation), HIE (N_ϵ protonation), and HIP (N_ϵ and N_δ protonation) (see Fig. 1 *C*). Mutation of the stacking His in an AA9 from *Myceliophthora thermophila* (referred to as *Mt*PMO3*) resulted in a slower turnover of oxygen (in terms of H_2O_2 production rate) relative to the wild type, showing that this conserved residue is important for the oxidase activity of the enzyme (30). Of note, in the crystal structure of *Mt*PMO3* obtained at pH 3.9 (PDB: 5UFV) (30), an alternative rotamer of the stacking His that is rotated away from the histidine brace was observed in two out of four protein monomers in the asymmetric unit. Furthermore, in two instances, neutron diffraction structures of the AA9D from *Neurospora crassa* OR74A (*Nc*AA9D) indicated that the most likely protonation state for the stacking histidine is HIE at pH 5.6 (36,37). A similar observation was made for an AA9 LPMO from *Lentinus similis*, namely *Ls*AA9A (38). Based on a detailed analysis of stacking His side chains in several LPMOs after unrestrained crystallographic refinement, Banerjee et al. also concluded that the HIE form is the most populated state (39). Importantly, in addition to AA9 LPMOs, the stacking histidine is also found in bacterial cellulose-active C1/C4-oxidizing AA10s (40) and fungal AA14s (41). It has been noted that, at low pH, one of the copper-coordinating residues in AA9 LPMOs tends to become disordered, a phenomenon that may be attributed to the protonation of this residue (42).

Here, we present computational data that shed light on the pH midpoint titration values and protonation states of the stacking His residues in two different, well-characterized LPMOs: the C1-oxidizing AA9D from *Phanerochaete chrysosporium* RP-78 (*Pc*AA9D) and the C4-oxidizing *Nc*AA9D. We examined the properties of the stacking His residues using Gaussian accelerated and replica-exchange constant-pH molecular dynamics (MD) simulations and density functional theory (DFT). The effect of substrate binding on stacking-His ionization behavior was also assessed. Our results are in line with previous experimental observations, the molecular origin of the pH-dependent orientation of the stacking histidine is revealed, and we discuss how our results provide new insight into the mechanistic aspects related to O_2 activation and H_2O_2 stabilization during LPMO catalysis.

MATERIALS AND METHODS

Construction of the initial models

Inputs for the MD simulations and free-energy calculations were obtained from the PDB entries 4B5Q, *Pc*AA9D from *Phanerochaete chrysosporium* RP-7 (43), and 4EIR, *Nc*AA9D (also called *Nc*PM O -2) from *Neurospora crassa* OR74A (44). The models were prepared by manual editing and the program pdb4amber program in the AmberTools23 package (45). The protonation state of each titratable amino acid side chain was predicted at pH 6.0 using H++ (v3.2) software package (46), and the input PDB files was updated accordingly. Disulfide bridges were identified for Cys resides 43 and 163 in *Pc*AA9D and for 39 and 171, and 141 and 223 in *Nc*AA9D. The program tleap (AmberTools23) was used to solvate the proteins in TIP3 water molecules using a box size that ensured 14 Å of solvent around the enzymes. Sodium ions were added to the *Pc*AA9D models and chloride to the *Nc*AA9D models to ensure charge neutrality during the simulations.

The cellulose model was built using the program cellulose builder (47) and consisted of five layers of 24× glucose chains distributed as 3:4:5:6:5 (from lower to upper layer) in a diamond shape (see Fig. S1, gray dotted line). The *Pc*AA9D was oriented on the cellulose crystal as described by Wu et al. (43), and the histidine brace was positioned relative to the C1 exactly as in the previously published *Sm*AA10A model on β-chitin (35). Two starting models (model A and B) were built where the substrate-binding surface-exposed Tyr75 displayed two different rotamers.

For all the simulations, the AMBER ff14SBforce field was used for the protein (48), the GLYCAM_06 (49) was used for the cellulose, and the Joung/Cheatham ion parameters for TIP3P (50) were used for water and ions. In addition, parameters for both Cu(I) and Cu(II) versions were determined, using the *Pc*AA9D active site including the copper ion, His1, His76, Gln158, and Tyr160, as a starting model. In short, fragment-restrained geometry optimizations followed by frequency analysis of the Cu(I) and Cu(II) form of the active-site models were carried out in ORCA4 (51) using the B3LYP hybrid functional (52) and the cc-pVDZ basis set (cc-pVTZ for copper) (53). For the Cu(I) model, only the amino acids were included in the model, whereas, in the Cu(II) model, a copper-binding water molecule was added to retain the active-site geometry. The final force-field parameters were then calculated using the in-house program PyParam (https://github.com/kjendseth/PyParam), which integrates the calculation of the electrostatic potential (ORCA, orca_vpot) (51), RESP charges (AMBER, respgen) (45), and our implementation of the Seminaro method (54). While testing the force-field parameters, we found that the active-site integrity was best maintained using the force constants calculated for the Cu(II) active-site model for both Cu(I) and Cu(II) AA9 force-field parameter sets. The redox state differences in our force-field parameters were reflected by different partial charges and a slightly shorter Tyr-O–Cu bond for the Cu(II) force-field parameter set. The developed AA9 force-field parameter sets are listed in Table S1.

Initial MD simulations

For all models, the first stage was a 5000-step energy minimization performed on the entire system. During the minimization, the nonhydrogen atoms of the enzymes were positionally restrained with a harmonic potential of 10 kcal mol^{-1} Å^{-2} (restraints are explicitly mentioned in the steps where they are applied). Then, the systems were heated linearly from 0 to 300 K for 40 ps at constant volume, with restraints lowered to 1 kcal mol^{-1} Å^{-2}, using the Langevin thermostat with a collision frequency of 1 ps^{-1}. Density equilibrations were run at 300 K for 0.5 ns at a constant pressure of 1 atm using the Berendsen barostat with a pressure relaxation time of 1 ps. The final 100-ns equilibration step was carried out in the NVT ensemble using the weak coupling algorithm and a time constant of 10 ps to regulate the temperature. In all simulations, we used 2-fs time steps, periodic boundary conditions with a 12-Å cutoff for nonbonded interactions, and PME treatment of long-range electrostatics (55), whereas hydrogen atoms were constrained by the SHAKE algorithm (56). Simulations were carried out using the CUDA version of PEMMD included in AMBER22 (57). Analysis of production trajectories was performed using the *cpptraj* module included in AmberTools23 (58).

The protocol for the *Pc*AA9D-cellulose complexes (model A and B) was slightly different. After the energy-minimization stage, the C1 atoms in the cellulose crystal were positionally restrained by a harmonic potential of 2 kcal mol^{-1} Å^{-2} for 50 ns to equilibrate intra-crystal interactions. In the following 500-ns equilibration stage (NVT settings as mentioned above), the C1 atoms not in the top cellulose layer were restrained by harmonic

potential of 1 kcal mol^{-1} Å^{-2}, and the H1 (H atom to be abstracted)-Cu distance was restrained to 3.8 Å by a harmonic potential of 5 kcal mol^{-1} Å^{-2}. This system maintained a histidine-brace positioning on the substrate closely resembling the *Sm*AA10A model on β-chitin, which has been predicted to be catalytically active (59).

Gaussian accelerated MD simulations

To explore a wider conformational space more efficiently than with conventional MD simulations, we performed Gaussian accelerated MD (GaMD) simulations, which is an enhanced sampling technique that lowers energy barriers (60). Models of *Pc*AA9D and *Nc*AA9D, each in the Cu(I) and Cu(II) redox states, and with the stacking histidine (His) in the HIE, HID, or HIP protonation states, were equilibrated for 100 ns before being subjected to GaMD simulations.

The simulations were performed in the NVT ensemble with the parameters described above for 1 μs each, with the following GaMD-specific simulation flags: igamd = 3 (dual boost on both dihedral and total potential energy), iE = 1 (threshold energy set to the lower bound), ntcmdprep = 800,000 (number of preparation conventional MD steps), ntebprep = 800,000 (number of preparation biasing MD simulation steps), ntcmd = 400,0000 (number of initial conventional MD simulation steps), nteb = 4,000,000 (number of biasing MD simulation steps), ntave = 200,000 (number of simulation steps used to calculate the average and standard deviation of potential energies), sigma0P = 6.0 (upper limit of the standard deviation of the first potential boost), and sigma0D = 6.0 (upper limit of the standard deviation of the second potential boost). Coordinates were recorded every 1000 steps, resulting in trajectories with 500,000 frames for each simulation. The trajectories were analyzed using cpptraj (58), the values for the dihedral angles χ_1 and χ_2 were extracted for each frame, and the data were reweighted using the program PyReweighting (github.com/MiaoLab20/pyreweighting) by cumulant expansion to the second order (61,62).

Constant-pH MD simulations

The stacking His in *Pc*AA9D and *Nc*AA9D was examined by constant-pH MD simulations (63) with both the Cu(I) and Cu(II) force-field parameter sets. Additionally, two starting models of *Pc*AA9D on cellulose were investigated with the Cu(I) force-field parameter set. The difference between the two *Pc*AA9D-cellulose models was the rotamer of the Tyr75 residue that either pointed toward the copper ion or away from the copper ion (see Fig. S1). The total equilibration time for AA9s in solution and AA9 with cellulose before starting the constant-pH simulations were 100 and 500 ns, respectively. When running the constant-pH simulations with the AA9-cellulose complex, an additional set of weak positional harmonic restraints of 0.5 kcal mol^{-1} Å^{-2} were applied to the protein C_α atoms in addition to the C1 restraints on cellulose and the Cu-H1 restraint to ensure overall stable ensembles. All explicit solvent constant-pH simulations were run applying a salt concentration of 0.1 M, 100 steps between protonation attempts, and 200 steps of solvent relaxation for each attempt. For each model, we ran 16 parallel 200-ns replica-exchange simulations at pH 1.5–9.0 (0.5-unit intervals) and replica exchange was attempted every 1000 steps. The data were reordered using the program cphstats included with AmberTools23. Eq. 1 was applied when fitting the Henderson-Hasselbalch equation to the data (f_d is fraction of protonated HIP state).

$$f_d = \frac{1}{\left(1 + 10^{(n \bullet (pK_a - pH))}\right)} \qquad \text{(Equation 1)}$$

DFT calculations

The *Pc*AA9D active-site model was derived from the 4B5Q crystal structure (43), and partly the structure of *Ls*AA9A, PDB: 5N04, when building models of displaced His76 (42). The models were geometry optimized using the BP86 (64,65) and B3LYP (52) DFT functionals and the Def2-SVP basis set, applying the larger Def2-TZVPP basis set at the copper ion (53), including the D4 dispersion correction with Becke-Johnson dampening (66). Final single point energies were calculated using the B3LYP functional and the Def2-TZVPP basis set on all atoms. To ensure that the active-site model resembled the crystal structure active site, we applied a fragment-based restraining scheme that is available in ORCA. Each amino acid, the copper ion, and water molecules (if present) were described as individual fragments. Amino acid-containing fragments were connected at the C_α that was saturated with hydrogen atoms to form a methyl group. An example of an ORCA input files can be found in the supplementary information, and geometry-optimized coordinates are provided as a zip file.

RESULTS

Classical MD simulations

Using MD simulations, we examined the active-site integrity of *Pc*AA9D and *Nc*AA9D with the stacking His in different protonation states using our AA9 force-field parameters (Table S1). The active-site average structures of the initial 100-ns MD trajectories of solvated *Pc*AA9D (PDB: 4B5Q) (43) and *Nc*AA9D (PDB: 4EIR) (44) with the stacking His in the HID, HIE, and HIP protonation states were compared with their respective crystal structures (Fig. S2). For both enzyme models, the HIE protonation state assumes a rotamer conformation that resembles that observed in their respective crystal structures, whereas the HID state displays higher disorder of the side-chain atoms. When the stacking His is modeled in the HIP state in the two enzyme models, the side chain is rotated around the C_α–C_β bond (χ_1) and adopts a solvent-exposed "outward" conformation (see dark gray structure in both Fig. 2 *A* and *B*). This solvent-exposed conformation of the HIP side chains resembles the conformation of the stacking His observed in the crystal structure of *Mt*PMO3* (PDB: 5UFV), which was crystalized at pH 3.9 (see pink and purple structures in Fig. 2) (30).

Gaussian accelerated MD simulations of *Pc*AA9D and *Nc*AA9D in solution

To investigate the effect of the stacking His protonation state on its side-chain geometry, we performed 1-μs simulations of both *Pc*AA9D and *Nc*AA9D in solution with the stacking His in either the HIE, HID, or HIP state with the copper ion in either the Cu(I) or Cu(II) state. The analysis of the trajectories and the reweighted energies are shown in Fig. 3. The dihedral angles χ_1 and χ_2 (see Fig. 1) are plotted on the x and y axis, respectively, and the corresponding potential of mean force is indicated in kcal/mol. The deep red color indicates high-energy states that are not likely to be populated, whereas blue color suggests more energetically favorable states. The χ_1 and χ_2 dihedral angles measured in the crystal structures of *Pc*AA9D (Fig. 3 *A*, −70.7°, 119.9°) and *Nc*AA9D (Fig. 3 *B*, −64.8°, 100.7°) are indicated by magenta circles. Although the potential energy surfaces of

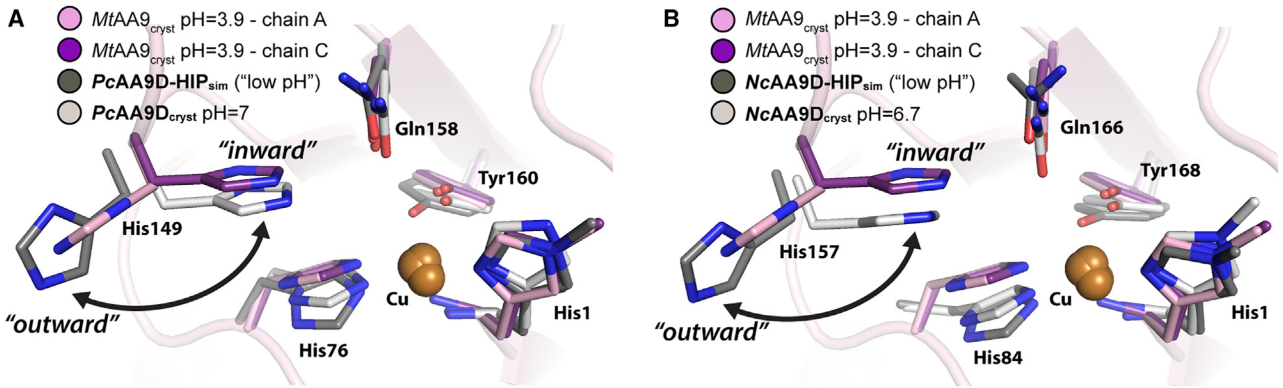

FIGURE 2 Comparison of crystal structures and modeled AA9 active sites at different pH values. In both (*A*) and (*B*), the active site of *Mt*PMO3* (PDB: 5UFV) is shown with the stacking His oriented away (pink, chain A) and toward (purple, chain C) the copper site. (*A*) Comparison of the model of *Pc*AA9D with the stacking His in the HIP state (*Pc*AA9D-HIP$_{sim}$, dark gray, obtained after 100 ns of MD simulation) with the crystal structure of *Pc*AA9D (*Pc*AA9D-$_{cryst}$, PDB: 4B5Q, light gray). (*B*) Comparison of the corresponding model of *Nc*AA9D (*Nc*AA9D-HIP$_{sim}$, dark gray, obtained after 100 ns of MD simulation) with the crystal structure of *Nc*AA9D (*Nc*AA9D$_{cryst}$, PDB: 4EIR, light gray). To see this figure in color, go online.

HID and HIP forms of both LPMOs show an archipelago of populated states, the HIE forms are found in a more restricted area that corresponds to the conformation observed in the crystal structures (the magenta circle overlays with the low energy blue area). It can also be observed that the distribution of low-energy wells is slightly wider for the Cu(I) oxidation states in the HIE form than the Cu(II) oxidation states. This may indicate that the water molecules coordinating to the Cu(II) state (and to a lesser extent to the Cu(I) state due to the lower partial charge of the Cu in this state) influence the conformation of the stacking His in the HIE state.

Constant-pH simulations of LPMOs in solution reveal a pH-dependent conformation of the stacking His

To estimate the pK_a values of the stacking His in *Nc*AA9D and *Pc*AA9D in water, we employed replica-exchange constant-pH simulations (67). Using this method, we could determine the fraction of the doubly protonated HIP state in the pH range 1.5–9.0. The effect of the force field was also investigated by performing simulations with both the Cu(I) and Cu(II) parameter sets that were developed for each redox state (Table S1). In each simulation, 16 replicas $\times$ 200 ns representing different pH values were evaluated, and the resulting titration curves (in blue) are shown in Fig. 4. The apparent pK_a values (referred to as midpoints of the pH titration throughout the paper) for the stacking His in *Nc*AA9D and *Pc*AA9D display similar trends. When inspecting the fraction of the HIP state of the stacking His at different pH values during the simulation, it became clear that the curves were fluctuating around the apparent pK_a values (Fig. S3). This observation aligns well with the results from the GaMD simulations that indicate that the rotamer conformation of the stacking His depends on the protonation state. The trajectories that were simulated at the different pH values were examined, and there are clearly two well-separated populations of the stacking His rotamer being formed around χ_1 values of $-74°$ and 60°, and the size of the populations depends on the pH (Figs. S4 and S5). These two populations represent the "inward" and "outward" rotamer of the stacking His (see Fig. 2), the fractions of which were plotted against the pH value (Fig. 4, orange lines). There is a clear correlation between the fraction of HIP calculated from the Monte Carlo sampling in the constant-pH simulations and the conformation of the outward rotamer, showing that, when protonated to HIP, the stacking His rotates to the outward conformation. This is confirmed by fitting the Henderson-Hasselbalch equation to the dataset results, which show the same titration midpoints.

The confining environment of the stacking His determines its rotamer conformation and ionization properties

Considering the potential importance of the stacking histidine residue in the LPMO catalytic mechanism, we estimated the effect of LPMO substrate binding on the pH-dependent behavior of this residue. Given the availability of both experimental and computational data supporting the reliability of models of C1 oxidizers in complex with crystalline substrates (35,43), this part of the study focused on a C1 oxidizer. The initial complex of the C1-oxidizing *Pc*AA9D and I_β-cellulose was constructed adopting the orientation of the enzyme on the cellulose surface previously reported in modeling studies on *Pc*AA9D binding to I_β-cellulose (43). After 500 ns of MD simulation, the *Pc*AA9D-I_β-cellulose complex appeared stable, and the surface-exposed aromatic residues, Tyr28 and Tyr198, were observed to be aligned with the cellulose chains (Fig. S1). Of note, these residues have previously been shown to interact with the hydrophobic surface of the cellulose fiber

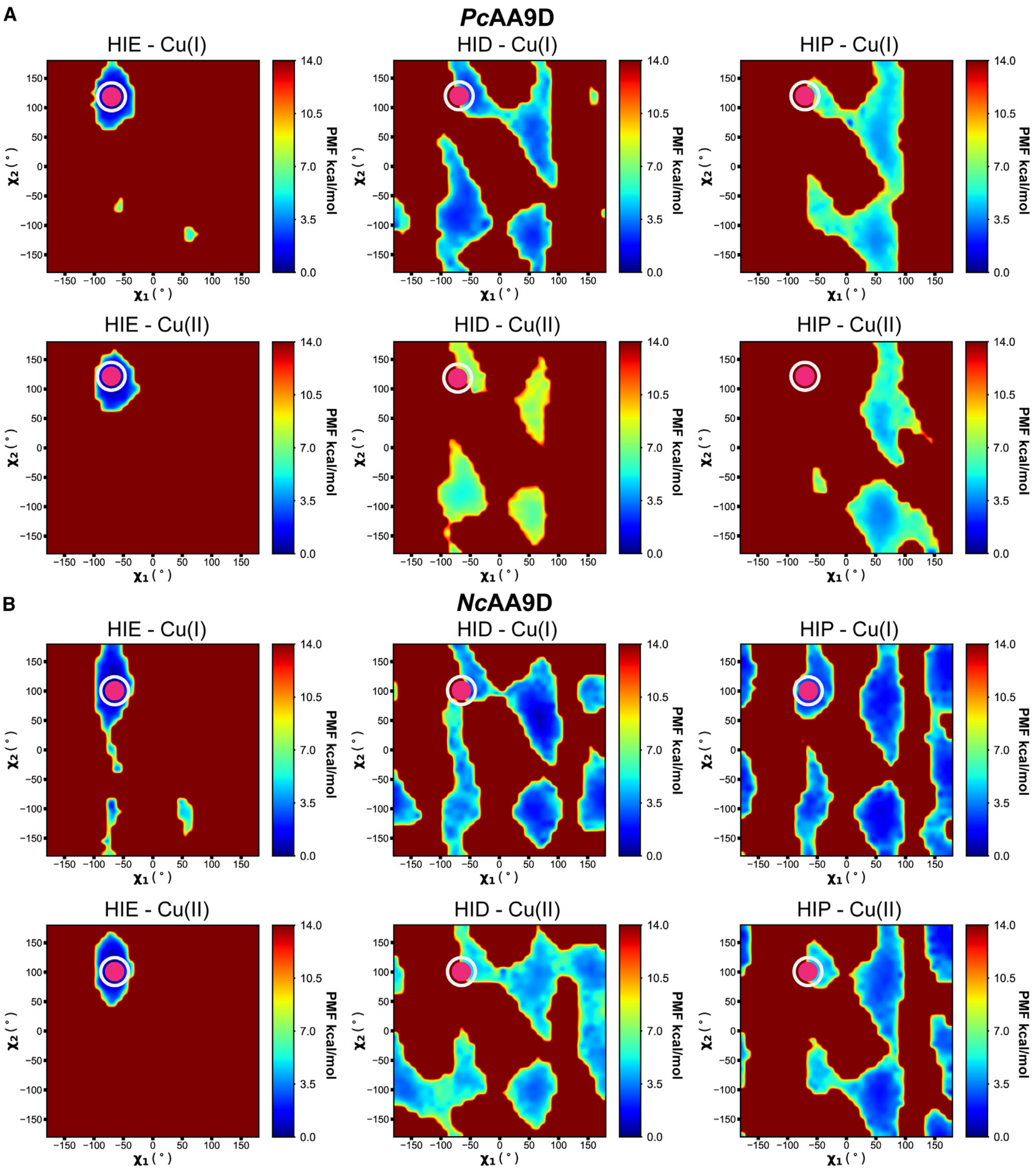

FIGURE 3 Energetics of stacking His conformations probed by Gaussian accelerated MD simulations. The potential energy surfaces as functions of the side-chain dihedral angles χ_1 and χ_2 for the stacking His HIE, HID, and HIP states for (*A*) *Pc*AA9D and (*B*) *Nc*AA9D. In each panel, the upper and lower plots correspond to simulations with the active-site copper in the Cu(I) and Cu(II) oxidation state, respectively. The magenta circles indicate the experimental values of χ_1 and χ_2 in the crystal structures for *Pc*AA9D (PDB: 4B5Q) and *Nc*AA9D (PDB: 4EIR). For comparison, the dihedral angle χ_1 of the outward conformation of the stacking His in the crystal structure of *Mt*PMO3* (PDB: 5UFV) is 60°. To see this figure in color, go online.

(43). The stacking His did not form any hydrogen bonds to the cellulose substrate in any of our simulated models. The His-brace and copper positioning of *Pc*AA9D relative to the substrate closely resemble that previously observed for the *Sm*AA10A-β-chitin complex (35). Of note, such geometry is likely to be coherent with efficient catalysis (19–21).

In the constant-pH simulations, we monitored transitions between the HID, HIE, and HIP states of the stacking His in

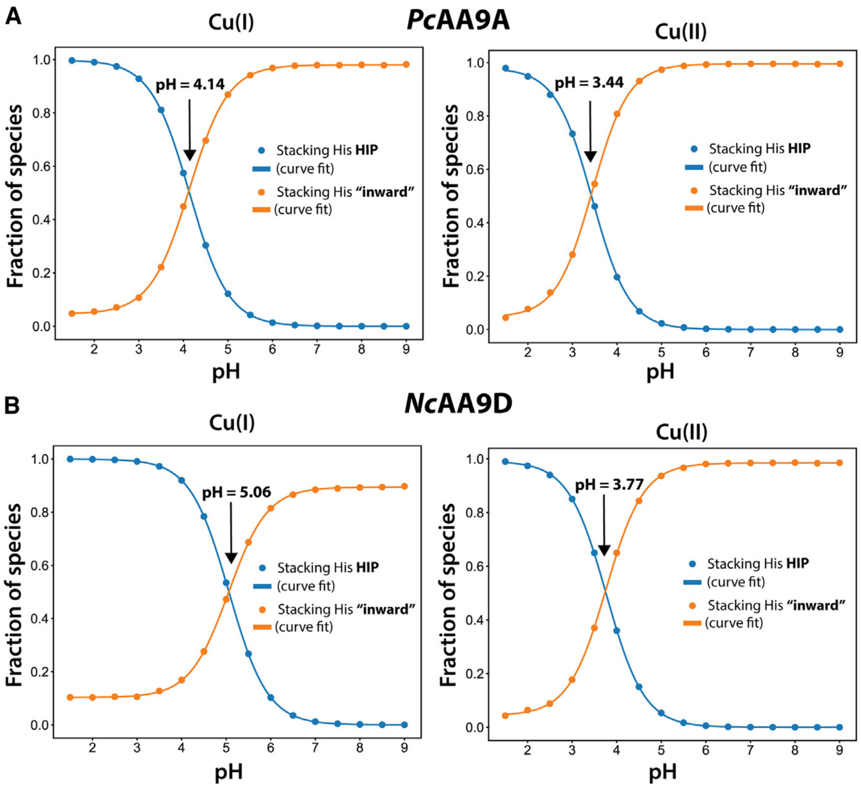

FIGURE 4 Estimation of titration midpoints of the stacking His by constant-pH MD simulations. The fraction of the protonation state HIP (in blue) and the fraction of stacking His with the "inward" rotamer (in orange at pH 1.5–9.0; 16 equally spaced pH values) for (*A*) *Pc*AA9D and (*B*) *Nc*AA9D, computed with Cu(I) (left panels) and Cu(II) (right panels) active-site force-field parameters is indicated. The data (filled circles) were fitted (solid line) using Eq. 1 and black arrows indicate titration midpoints. To see this figure in color, go online.

the LPMO-cellulose complex. In simulations without cellulose, *Pc*AA9D has a surface-exposed Tyr residue (Tyr75) close to the copper site that displays two different rotamers that was modeled when on cellulose in model A and B, respectively. The effect of these rotamers in starting models A ($\chi_1 = -64.7°$) and B ($\chi_1 = 160.5°$) on the HIP-HIE/HID equilibrium was investigated. We observed few transitions between the HID and HIE states (~0.5% HID), and the HIP state was rarely observed for both models A and B (see statistics in Table S2). When examining the trajectories for both model A and B, it appears that only one of the χ_1 rotamers is populated for both models (Fig. S6).

The confined cavity that forms upon association of the LPMO with the polymeric substrate is partly protected from the aqueous environment, and the stacking His side chain is not allowed to rotate around the C_α–C_β bond (χ_1) and enter solution (outward rotamer) because it is physically restrained by the cellulose substrate and the residues Val150, Pro77, and His76 (Fig. 5 *A* and *B*). When examining our equilibrated MD models and the crystal structures, it was found that the HID (and thus HIP) protonation state is energetically unfavorable due to the severe steric clash that will happen when the stacking His is in the inward position (Fig. 5 *C*). Thus, in contrast to what we observed in solution (vide supra), cellulose binding by *Pc*AA9D restricts the stacking His to the HIE protonation state, regardless of the pH value of the solution (tested from 1.5 to 9.0).

Alternative protonation of the copper-coordinating internal copper-coordinating histidine

Based on a crystallographic study it has been suggested that the internal His that binds copper and is part of the histidine brace can be protonated before the stacking His (42). When this internal His residue becomes protonated, it is no longer be able to coordinate copper, and it takes a conformation that may destabilize the LPMO active site. The classical MD simulations performed in this work are not suitable to access such a scenario because it involves the breaking and formation of bonds. Instead, we built, geometry optimized, and compared an array of relevant *Pc*AA9D models using DFT, with copper in the Cu(I) (Fig. 6 *A*) and Cu(II) (Fig. 6 *B*) states. Of note, the Cu(II) ion coordinates two water molecules (readily observed in LPMO crystal structures that are not severely photoreduced) and the equatorial water molecule is hydrogen bonded to Gln158 (Fig. 6 *B*). The Gln158 side chain may adopt a variety of rotamer configurations. To corroborate this possibility, the MD simulation trajectories of models featuring the stacking His residue in the HIE state with both Cu(I) and Cu(II) in the active site were investigated. The distributions of the Gln158 side-chain dihedrals χ_1 to χ_3, along with the distance between the Tyr160-OH HH atom and the Gln158 OE1 atom, are presented in Fig. S7. The observed dihedrals are distributed around those values measured in the crystal structure,

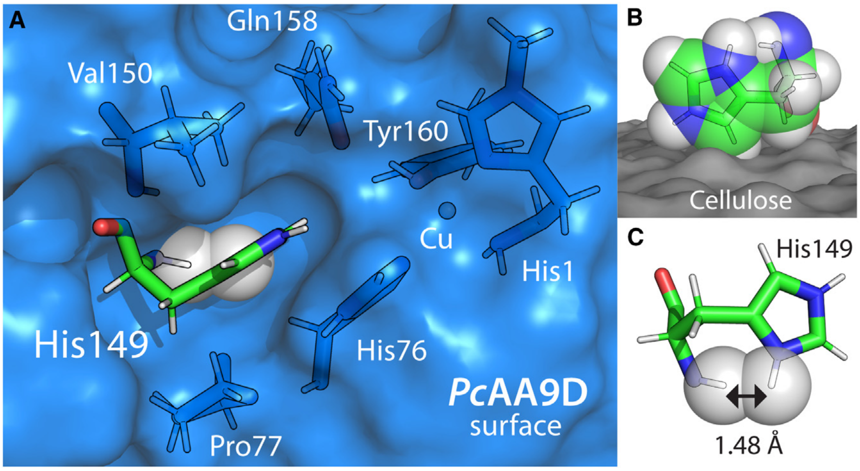

FIGURE 5 Confinement of the stacking histidine. (*A*) The stacking His149 (shown as green stick) is partly buried in a pocket lined by the side chains of the hydrophobic residues Val150 and Pro77 and the copper-coordinating His76. The model is taken from a snapshot after 500-ns equilibration of *Pc*AA9D on a cellulose model (gray surface). The stacking His side chain is shown in the HIP state, in inward position (the N_δ-H is ignored during the simulation), and the N_δ-H and the amide backbone hydrogen are shown as gray spheres (van der Waals radius applied). (*B*) Same model as in (*A*), where the cellulose is shown as dark gray surface and all protein residues except the stacking His are hidden. (*C*) Close-up view of the stacking His in the same model showing that, when it is protonated at the N_δ, there is a severe steric clash with the amide hydrogen (expected distance around 2.2 Å). To see this figure in color, go online.

indicating that the Gln158 side chain have limited flexibility. This may be explained by frequent hydrogen-bonding interactions with the Tyr160.

To address the possible protonation of the internal His residue, a *Pc*AA9D-modified active-site model, where the His76 was moved to the position observed for the corresponding displaced residue in *Ls*AA9A (42), was built (Fig. 6 *C*). The ring of the displaced His can possibly take two orientations, and these were modeled as mode 1 and mode 2. In Fig. S8, the active-site models of *Pc*AA9D with the His76 in displaced modes (gray carbons) are compared to models where His76 coordinates to the copper ion (magenta carbons). No models of the stacking His in the HID state were included since the inward rotamer of His149 is not compatible with these states in the enzyme. However, active-site models featuring the stacking His in the HIP state were used as references to enable energy comparisons of isomeric models (His76 vs. His149 in the HIP state) where the steric clash is eliminated when capping the side chain at C_α. The DFT calculations suggest that the models with displaced His have significantly higher energy than the copper-coordinating models, both for Cu(I) ($\Delta E \sim 25$ kcal/mol) and Cu(II) ($\Delta E \sim 32$ kcal/mol) states (see Fig. S8).

Activation and stabilization of O_2 in the typical AA9 active site

The stacking His residue is positioned close to the copper ion that is bound in the AA9 active sites. In *Pc*AA9D the distance between the copper ion and the N_ε of the stacking His is only 4.8 Å, indicating that small-molecule ligands that bind to copper in the equatorial plane, which is defined by the copper ion and the three N atoms that coordinate the metal, can interact with the stacking His. To illustrate how the stacking His can influence O_2 binding to the Cu(I) state, the active-site model of *Pc*AA9D in the Cu(I) state was modified to also include molecular oxygen. The triplet state reflecting a ferromagnetically coupled Cu(II)–superoxide complex was 3.6 kcal/mol lower in energy than the singlet, antiferromagnetically coupled state. In the geometry-optimized model, the hydrogen atom at the N_ε position of the stacking His forms a hydrogen bond to the distal O atom of the superoxide molecule (Fig. 6 *D*). The results also indicate that an H_2O_2 molecule near the position of the bound superoxide will also be in hydrogen-binding range with the stacking His, which may help orient this co-substrate molecule.

DISCUSSION

Our main objective in this study was to map the chemical properties and shed light on the function of a highly conserved His residue found in the second coordination sphere of AA9 LPMOs. Important properties such as pK_a values and the preferred protonation states of this His residue are difficult to assess by experimental methods. Previously, the AA9 reaction mechanism has been investigated using computational methods (19,20,43,68,69), and AA9-related reaction schemes have been suggested with limited knowledge on the favored protonation state of the stacking His. Here, we discuss our results and their implications for AA9-catalyzed O_2 and H_2O_2 activation.

Both white-rot and brown-rot fungi secrete AA9 enzymes when depolymerizing lignocellulosic biomass (70). Typical white-rot fungi encode many more AA9 enzymes in their genomes than brown-rot fungi (16 vs. 4 genes on average, respectively (71)); it is, however, not clear if these types of wood-decaying fungi utilize LPMOs in the same way. LPMOs from both brown-rot and white-rot fungi likely enter an acidic environment when secreted from the host hyphae. The fungi are known to produce organic acids such as oxalic acid and lower the pH in their environment—brown-rot species more so than white-rot species. Healthy wood has a slightly acidic pH (around 5–6), depending on the species (72). In deadwood, where the microbial communities that decay lignocellulose can be found, the overall pH may be in the range of 3.5–5, perhaps even lower in some microenvironments (73). Notably, the pH optima of

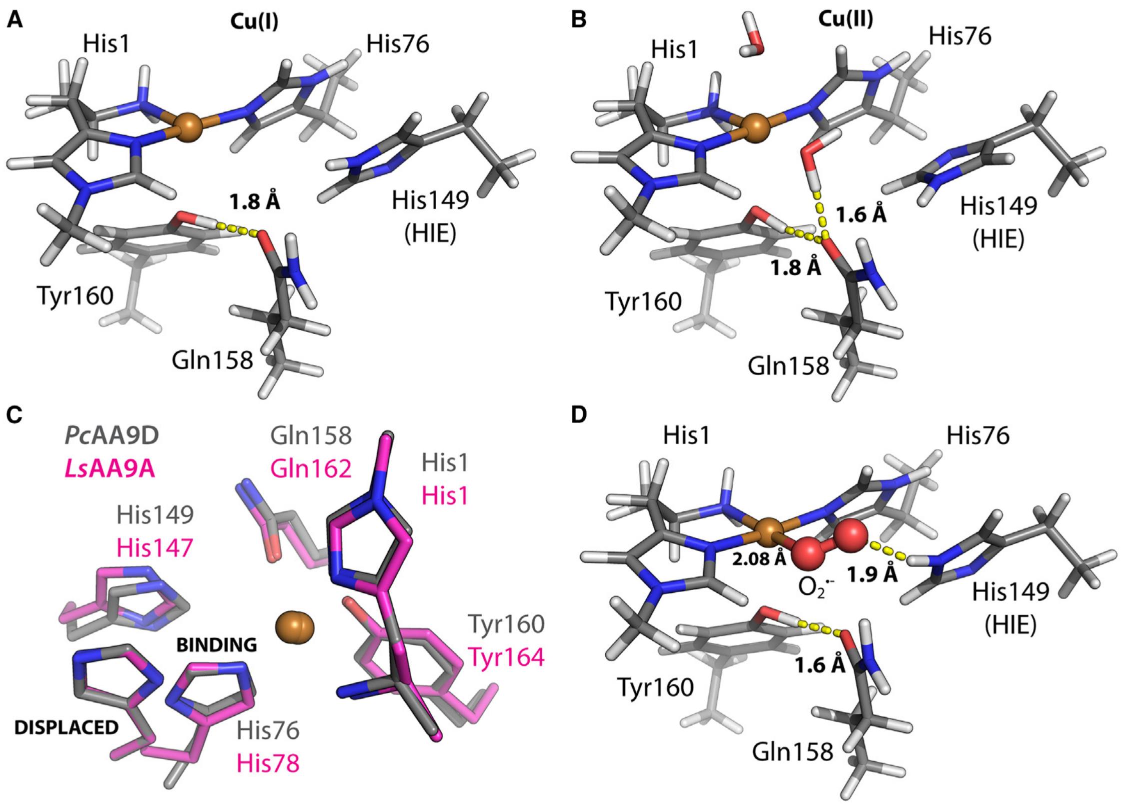

FIGURE 6 Geometry-optimized active-site models of *Pc*AA9D. DFT geometry-optimized model (B3LYP) of the *Pc*AA9D active site, in solution (i.e., no cellulose), with the stacking His149 in the HIE state, for (*A*) the Cu(I) and (*B*) the Cu(II) states. In neither model can the stacking His form hydrogen bonds to other amino acid side chains or copper-coordinating water molecules. (*C*) Superimposed active sites of *Pc*AA9D and *Ls*AA9A. In the *Ls*AA9A structure 5N04, the His78 (corresponding to His76 in *Pc*AA9D) is displaced and no longer coordinates to the copper ion. To mimic this scenario in our *Pc*AA9D (gray carbons) active-site models, the His76 was translated and rotated into the position observed in *Ls*AA9A (magenta carbons). (*D*) The Cu(II)-superoxide complex with the stacking His in the HIE state in DFT geometry-optimized model of the *Pc*AA9D active site, in solution (i.e., no cellulose). The stacking His in the HIE state is shown with the superoxide hydrogen bonded to the N_ϵ-H. Other polar second-sphere residues such as Tyr160 or Gln158 are not predicted to form hydrogen bonds with superoxide. The copper atom is shown as an orange sphere. To see this figure in color, go online.

hydrolytic cell-wall-degrading enzymes are often found to be around 5 (74), and one of the most popular commercial LPMO-containing cellulose-degrading enzyme cocktails (CTec2, Novozymes) has a recommended pH range of 5–5.5 for efficient saccharification. In light of this knowledge, it is crucial to understand the effect of pH on the stacking His in AA9-LPMOs and how slightly acidic pH can alter the LPMO active-site properties and, thus, its mode of action.

We discovered that the side chains of the stacking His in *Nc*AA9D and *Pc*AA9D exhibit a highly pH-dependent conformation when in solution in the absence of substrate. The GaMD simulations shown in Fig. 3 allow for efficient sampling of the energy landscape, and it is clear that the only protonation state that resembles the conformations observed in the crystal structures are those with the stacking His in the HIE protonation state. To increase the resolution of the pH dependency and determine at which pH values the conformational transition of the stacking His occur, we carried out a series of constant-pH simulations. Our data clearly show that there is a correlation between the simulation pH and the conformation of the stacking His. This was confirmed by analyzing both results from the Monte Carlo protonation attempts and the populations of the stacking His rotamers in the trajectories. A rotamer orientation where the stacking His side chain points toward the active-site copper was found to become the major orientation at pH levels above approximately 3.5 for the Cu(I) state and between 4 and 5 for the Cu(II) state (Fig. 4). At pH values lower than these thresholds, steric clashes between the hydrogen of the protonated N_δ of the stacking His and the amide proton of the same residue within the confined protein structure force the side chain to rotate into an outward conformation, making it more exposed to the solvent (Fig. 5). Although an analysis of the conservation scores of the amino acids lining the stacking His pocket indicate that the Val150 and Pro77 (Fig. S9) are not conserved, the dihedral angles ϕ and χ_1 of the stacking His (Fig. S9) that are consistent with an energetically unfavorable inward conformation for the HID and HIP states are observed in all the available AA9 crystal structures (Fig. S10). The different behavior we observed for the Cu(I) and Cu(II) force-field parameter sets can be

explained by the different partial charges distributed on the His-brace residues, the copper ion, and the buried Tyr residue. The higher partial charge of 0.5 for the Cu(II) ion versus 0.1 for Cu(I) ion will attract water molecules to the divalent active site and, to a larger extent, result in water binding to the copper site. This phenomenon has also been observed in simulations of AA10 LPMO models (35). Water molecules that interact electrostatically with the active-site copper in our simulations will also form hydrogen bonds to the stacking His side chain, thus affecting the midpoint values of the simulated pH titrations.

Our data are consistent with those observed in the crystal structure of *Mt*PMO3* (PDB: 5UFV) that was crystalized at pH 3.9 (30). In this structure, which consists of four enzymes in the asymmetric unit, the stacking His displays a rotamer pointing toward the copper site (as observed in our simulations of the HIE state) in two of the protomers. For the other two protomers, the stacking His resembles the conformation of the HIP side chain in our simulations, the side chain pointing out toward the solvent. The crystallographic data, where conformations of the stacking His are 50% HIP-like and 50% HIE-like at pH 3.9, may indicate a similar response to pH to what we predicted for *Nc*AA9D (midpoint of pH titration of 3.8) and *Pc*AA9D (3.4) (Fig. 4).

Altogether, our data indicate that, upon reduction of Cu(II) to Cu(I), a priming event necessary for LPMO catalysis to take place, a greater fraction of HIP, predicted to adopt a solvent-exposed conformation, is expected to be observed at acidic pH. One may speculate that movement of the stacking His between bulk solvent and the active site could be part of a proton shuttle that transports protons in and out of the active-site pocket. For reactions in solution, in the absence of polysaccharide substrate, proton transport into the active site could be important when copper-bound O_2 is reduced to superoxide/ H_2O_2. Interestingly, our simulations indicate that the conformation and protonation sate of the stacking His interchange rapidly at pH values close to the titration midpoint in solution (Fig. S3) and that this process occurs at a higher rate in the Cu(I) redox state of the active site. On the other hand, when the LPMO is bound to a cellulose surface, our calculations indicate that the conformation of the stacking His is restricted and that the only relevant protonation state is HIE (see Fig. 5; Table S2). On a more speculative note, one may wonder if the rotation out of the active site of the predominant HIP state of the stacking His at low pH can have any biological importance in light of the low-pH conditions encountered during biomass fungal decay. LPMOs reduced in solution are sensitive to oxidative inactivation (I path); we speculate that this conformational change, thereby changing the active-site proton network and reactivity, may prevent the enzyme from catalyzing uncontrolled and deleterious reactions. This hypothesis warrants further biochemical and in silico investigations. We also investigated other possible protonation events in the active site, where the internal copper-coordinating His76 (in *Pc*AA9D) could be protonated to an HIP state and thus be displaced as a metal ligand. The energetics of this process is highly unfavorable (larger than ~24 kcal/mol), and our data do not support such events.

In the past few years, studies have shown that H_2O_2 boosts the rate of polysaccharide oxidation by LPMOs and that H_2O_2 (PO path) is preferred over O_2 (MO path) as co-substrate (16–18). It is, however, clear that LPMOs can act as oxidases (O path) and activate O_2 in the absence of polysaccharide substrate and that the ultimate reaction product is H_2O_2 released in solution (26,75), which can in turn be used by substrate-bound LPMOs in a peroxygenase reaction. Therefore, we investigated how the protonation state of the stacking His would influence the first step of the O_2 reaction on the way to H_2O_2 production, which is the formation of the copper-superoxide complex, in our model system. Recently, stopped-flow fluorimetry experiments demonstrated that the isolated Cu(I) form of the bacterial LPMO *Sm*AA10A reacts very slowly, on the scale of tens of minutes, with O_2 (k = 3.3 $M^{-1}s^{-1}$) (21,35). This is in stark contrast with what has been reported for the AA9A from *Thermoascus aurantiacus* (*Ta*AA9A), which was re-oxidized to the Cu(II) form in seconds when the Cu(I) form was mixed with oxygenated buffer (75). Such a striking difference may be due to the stacking histidine since there is no equivalent in C1-oxidizing AA10s (such as *Sm*AA10A). In these AA10s, an acidic Glu residue can be found 5.7 Å from the active-site copper (Fig. 1 *B*), which is likely too far to sustain acid/base catalysis, compared to the N_ε of the stacking His that is closer (4.7 Å away from copper in *Pc*AA9D, and 5.1 Å for *Ta*AA9A, PDB: 2YET (4)). Furthermore, such role would require a significantly elevated pK_a value of the glutamate side chain. So far, whether this Glu residue found in AA10 can act as an acid/base catalyst remains to be studied. In the case of the AA9s, observation of fast reoxidation of Cu(I) into Cu(II) by O_2 indicates that the formed Cu(II)-superoxide complex (59,75), rather than undergoing dissociation and regeneration of O_2 and LPMO-Cu(I), followed a pathway where the Cu(II) is maintained. From there, two scenarios can be envisioned: either superoxide could be released, resulting in the LPMO–Cu(II) form and superoxide in solution, or the superoxide could be protonated and further reduced to a peroxide species while bound to copper. Our DFT calculations suggest that the copper-superoxide complex, with an HIE-stacking His, can form a hydrogen bond of 1.9 Å from the protonated N_ε to the distal oxygen of the superoxide moiety and stabilize the Cu(II)-superoxide complex (Fig. 6 *D*). Thus, the hydrogen bond provided by the HIE state of the stacking His in AA9-LPMOs to the distal O atom of superoxide may increase the lifetime and perhaps alter the reactivity of this complex. Interestingly, Karlin and co-workers have demonstrated that, in monocopper model complexes, an increase in H-abstraction reactivity is observed when the metal ligand also forms a hydrogen bond to the proximal O atom (i.e., the O atom closest to the copper ion) of the superoxide (76,77). An increased lifetime of the superoxide complex would increase the likelihood for a reducing equivalent

from, for example, a low-molecular-weight reductant such as ascorbate or a second LPMO in the Cu(I) state to further reduce the superoxide to a Cu(II)-peroxo species. We suggest that the role of the stacking His is to stabilize the Cu(II)-superoxo complex by hydrogen-bond donation in the process of O_2 activation in solution, arriving at a similar conclusion as Hedegård et al., who applied a different computational setup (QM/MM calculations) on the enzyme *Ls*AA9A, which can act on soluble substrates (20) and thus display a different activity profile than *Pc*AA9D.

CONCLUSIONS

We have studied the behavior of the highly conserved histidine that forms a stacking interaction with the histidine brace in two AA9-LPMOs, the C1-oxidizer *Pc*AA9D and the C4-oxidizer *Nc*AA9D, using complementary computational methods. Our data suggest that the orientation of the stacking histidine side chain is correlated with its protonation state, and that, in the HID and positively charged HIP states, it is oriented away from the active site. Analysis of available AA9 structures indicates that this observation can be generalized to other AA9 enzymes. The low values for the pH midpoints determined for the stacking His side chain indicate that these enzymes have adapted to the slightly acidic biological habitat where they operate. We also find that, at the standard pH used in most enzymatic studies (i.e., pH $\geq$ 5), the HIE state of the stacking His is favored when the enzymes are free in solution, and even more when bound to cellulose substrate. Finally, we suggest that the HIE state stabilizes the Cu(II)-superoxide species and that this can explain the efficient O_2 activation observed for fungal LPMOs relative to bacterial ones. Analysis of our results in light of the literature also supports the fact that the HIE state of the stacking histidine plays a crucial role in controlling the productive fate of otherwise damaging hydroxyl radicals generated along the peroxygenase reaction. Overall, our study pinpoints a phenomenon, namely the pH-dependent orientation and, thus, function of the stacking His, that should be considered when setting up LPMO reactions and interpreting their contribution, whether it be in mono-enzyme reactions or in secretome-like enzymatic cocktails, or for understanding their mode of action in biological processes.

SUPPORTING MATERIAL

Supporting Material can be found online at https://doi.org/10.1016/j.bpj.2024.04.002.

AUTHOR CONTRIBUTIONS

I.I. performed calculations, analyzed data, conceived experiments, and wrote the paper. S.J. analyzed data and wrote the paper. C.M.P. analyzed data, conceived experiments, supervised the project. and wrote the paper. B.B. analyzed data, conceived experiments, supervised the project, and wrote the paper. Å.K.R. performed calculations, analyzed data, conceived experiments, supervised the project, and wrote the paper.

ACKNOWLEDGMENTS

This work was supported by the Research Council of Norway grants 240967 and 301022 (Å.K.R.). S.J. and C.M.P. were supported by the National Science Foundation under grant no. 1552355. Computational work was performed on the Abel and Saga Clusters, provided by Sigma2 - the National Infrastructure for High-Performance Computing and Data Storage in Norway, project NN1003K/NS1003K; and the Extreme Science and Engineering Discovery Environment (XSEDE), which is supported by National Science Foundation grant number ACI-1548562, through allocation TG-MCB090159.

DECLARATION OF INTERESTS

The authors declare no competing interests.

REFERENCES

1. Aam, B. B., E. B. Heggset, …, V. G. H. Eijsink. 2010. Production of chitooligosaccharides and their potential applications in medicine. *Mar. Drugs.* 8:1482–1517.
2. Gelfand, I., R. Sahajpal, …, G. P. Robertson. 2013. Sustainable bioenergy production from marginal lands in the US Midwest. *Nature.* 493:514–517.
3. Langston, J. A., T. Shaghasi, …, M. D. Sweeney. 2011. Oxidoreductive cellulose depolymerization by the enzymes cellobiose dehydrogenase and glycoside hydrolase 61. *Appl. Environ. Microbiol.* 77:7007–7015.
4. Quinlan, R. J., M. D. Sweeney, …, P. H. Walton. 2011. Insights into the oxidative degradation of cellulose by a copper metalloenzyme that exploits biomass components. *Proc. Natl. Acad. Sci. USA.* 108:15079–15084.
5. Vaaje-Kolstad, G., B. Westereng, …, V. G. H. Eijsink. 2010. An oxidative enzyme boosting the enzymatic conversion of recalcitrant polysaccharides. *Science.* 330:219–222.
6. Forsberg, Z., G. Vaaje-kolstad, …, V. G. H. Eijsink. 2011. Cleavage of cellulose by a cbm33 protein. *Protein Sci.* 20:1479–1483.
7. Eibinger, M., J. Sattelkow, …, B. Nidetzky. 2017. Single-molecule study of oxidative enzymatic deconstruction of cellulose. *Nat. Commun.* 8:894.
8. Vermaas, J. V., M. F. Crowley, …, C. M. Payne. 2015. Effects of lytic polysaccharide monooxygenase oxidation on cellulose structure and binding of oxidized cellulose oligomers to cellulases. *J. Phys. Chem. B.* 119:6129–6143.
9. Villares, A., C. Moreau, …, B. Cathala. 2017. Lytic polysaccharide monooxygenases disrupt the cellulose fibers structure. *Sci. Rep.* 7, 40262.
10. Levasseur, A., E. Drula, …, B. Henrissat. 2013. Expansion of the enzymatic repertoire of the CAZy database to integrate auxiliary redox enzymes. *Biotechnol. Biofuels.* 6:41.
11. Johansen, K. S. 2016. Discovery and industrial applications of lytic polysaccharide mono-oxygenases. *Biochem. Soc. Trans.* 44:143–149.
12. Chylenski, P., B. Bissaro, …, V. G. Eijsink. 2019. Lytic Polysaccharide Monooxygenases in Enzymatic Processing of Lignocellulosic Biomass. *ACS Catal.* 9:4970–4991.
13. Beeson, W. T., C. M. Phillips, …, M. A. Marletta. 2012. Oxidative cleavage of cellulose by fungal copper-dependent polysaccharide monooxygenases. *J. Am. Chem. Soc.* 134:890–892.
14. Munzone, A., V. G. H. Eijsink, …, B. Bissaro. 2024. Expanding the catalytic landscape of metalloenzymes with lytic polysaccharide monooxygenases. *Nat. Rev. Chem.* 8:106–119.

15. Bissaro, B., A. K. Rohr, …, V. Eijsink. 2016. Fenton-type chemistry by a copper enzyme: molecular mechanism of polysaccharide oxidative cleavage. Preprint at bioRxiv. https://doi.org/10.1101/097022.

16. Bissaro, B., Å. K. Røhr, …, V. G. H. Eijsink. 2017. Oxidative cleavage of polysaccharides by monocopper enzymes depends on H2O2. *Nat. Chem. Biol.* 13:1123–1128.

17. Kuusk, S., B. Bissaro, …, P. Väljamäe. 2018. Kinetics of H 2 O 2 -driven degradation of chitin by a bacterial lytic polysaccharide monooxygenase. *J. Biol. Chem.* 293:12284–12531.

18. Hangasky, J. A., A. T. Iavarone, and M. A. Marletta. 2018. Reactivity of O 2 versus H 2 O 2 with polysaccharide monooxygenases. *Proc. Natl. Acad. Sci. USA.* 115:4915–4920.

19. Wang, B., E. M. Johnston, …, C. Rovira. 2018. QM/MM Studies into the H2O2-Dependent Activity of Lytic Polysaccharide Monooxygenases: Evidence for the Formation of a Caged Hydroxyl Radical Intermediate. *ACS Catal.* 8:1346–1351.

20. Hedegård, E. D., and U. Ryde. 2018. Molecular mechanism of lytic polysaccharide monooxygenases. *Chem. Sci.* 9:3866–3880.

21. Bissaro, B., B. Streit, …, Å. K. Røhr. 2020. Molecular mechanism of the chitinolytic peroxygenase reaction. *Proc. Natl. Acad. Sci. USA.* 117:1504–1513.

22. Filandr, F., P. Man, …, D. Kracher. 2020. The H2O2-dependent activity of a fungal lytic polysaccharide monooxygenase investigated with a turbidimetric assay. *Biotechnol. Biofuels.* 13:37.

23. Bissaro, B., E. Kommedal, …, V. G. H. Eijsink. 2020. Controlled depolymerization of cellulose by light-driven lytic polysaccharide oxygenases. *Nat. Commun.* 11, 890.

24. Costa, T. H. F., A. Kadic', …, S. J. Horn. 2020. Demonstration-scale enzymatic saccharification of sulfite-pulped spruce with addition of hydrogen peroxide for LPMO activation. *Biofuel. Bioprod. Biorefin.* 14:734–745.

25. Chang, H., N. Gacias Amengual, …, R. Ludwig. 2022. Investigating lytic polysaccharide monooxygenase-assisted wood cell wall degradation with microsensors. *Nat. Commun.* 13:6258.

26. Kittl, R., D. Kracher, …, R. Ludwig. 2012. Production of four Neurospora crassa lytic polysaccharide monooxygenases in Pichia pastoris monitored by a fluorimetric assay. *Biotechnol. Biofuels.* 5:79.

27. Breslmayr, E., M. Hanžek, …, R. Ludwig. 2018. A fast and sensitive activity assay for lytic polysaccharide monooxygenase. *Biotechnol. Biofuels.* 11:79.

28. Ciano, L., G. J. Davies, …, P. H. Walton. 2018. Bracing copper for the catalytic oxidation of C–H bonds. *Nat. Catal.* 1:571–577.

29. Gudmundsson, M., S. Kim, …, M. Sandgren. 2014. Structural and electronic snapshots during the transition from a Cu(II) to Cu(I) metal center of a lytic polysaccharide onooxygenase by x-ray photoreduction. *J. Biol. Chem.* 289:18782–18792.

30. Span, E. A., D. L. M. Suess, …, M. A. Marletta. 2017. The Role of the Secondary Coordination Sphere in a Fungal Polysaccharide Monooxygenase. *ACS Chem. Biol.* 12:1095–1103.

31. Vaaje-Kolstad, G., D. R. Houston, …, D. M. F. Van Aalten. 2005. Crystal structure and binding properties of the Serratia marcescens chitin-binding protein CBP21. *J. Biol. Chem.* 280:11313–11319.

32. Harris, P. V., D. Welner, …, L. Lo Leggio. 2010. Stimulation of lignocellulosic biomass hydrolysis by proteins of glycoside hydrolase family 61: Structure and function of a large, enigmatic family. *Biochemistry.* 49:3305–3316.

33. Loose, J. S. M., M. Ø. Arntzen, …, G. Vaaje-Kolstad. 2018. Multipoint Precision Binding of Substrate Protects Lytic Polysaccharide Monooxygenases from Self-Destructive Off-Pathway Processes. *Biochemistry.* 57:4114–4124.

34. Hall, K. R., C. Joseph, …, M. Sørlie. 2023. A Conserved Second Sphere Residue Tunes Copper Site Reactivity in Lytic Polysaccharide Monooxygenases. *J. Am. Chem. Soc.* 145:18888–18903.

35. Bissaro, B., I. Isaksen, Å. K. Røhr…, 2018. How a lytic polysaccharide monooxygenase binds crystalline chitin. *Biochemistry.* 57:1893–1906.

36. O'Dell, W. B., P. K. Agarwal, and F. Meilleur. 2017. Oxygen Activation at the Active Site of a Fungal Lytic Polysaccharide Monooxygenase. *Angew. Chem., Int. Ed. Engl.* 56:767–770.

37. Schröder, G. C., W. B. O'Dell, …, F. Meilleur. 2022. Capture of activated dioxygen intermediates at the copper-active site of a lytic polysaccharide monooxygenase. *Chem. Sci.* 13:13303–13320.

38. Tandrup, T., L. Lo Leggio, and F. Meilleur. 2023. Joint X-ray/neutron structure of Lentinus similis AA9_A at room temperature. *Acta Crystallogr. F Struct. Biol. Commun.* 79:1–7.

39. Banerjee, S., S. J. Muderspach, …, L. Lo Leggio. 2022. Protonation State of an Important Histidine from High Resolution Structures of Lytic Polysaccharide Monooxygenases. *Biomolecules.* 12:194.

40. Forsberg, Z., A. K. Mackenzie, …, V. G. H. Eijsink. 2014. Structural and functional characterization of a conserved pair of bacterial cellulose-oxidizing lytic polysaccharide monooxygenases. *Proc. Natl. Acad. Sci. USA.* 111:8446–8451.

41. Couturier, M., S. Ladevèze, …, J.-G. Berrin. 2018. Lytic xylan oxidases from wood-decay fungi unlock biomass degradation. *Nat. Chem. Biol.* 14:306–310.

42. Frandsen, K. E. H., J.-C. N. Poulsen, …, L. Lo Leggio. 2017. Unliganded and substrate bound structures of the cellooligosaccharide active lytic polysaccharide monooxygenase *Ls*AA9A at low pH. *Carbohydr. Res.* 448:187–190.

43. Wu, M., G. T. Beckham, …, M. Sandgren. 2013. Crystal structure and computational characterization of the lytic polysaccharide monooxygenase GH61D from the basidiomycota fungus Phanerochaete chrysosporium. *J. Biol. Chem.* 288:12828–12839.

44. Li, X., W. T. Beeson, …, J. H. D. D. Cate. 2012. Structural basis for substrate targeting and catalysis by fungal polysaccharide monooxygenases. *Structure.* 20:1051–1061.

45. Case, D. A., R. M. Betz, …, P. A. Kollman. 2016. Amber 2016. Univ. Calif. San Franc.

46. Anandakrishnan, R., B. Aguilar, and A. V. Onufriev. 2012. H++ 3.0: Automating pK prediction and the preparation of biomolecular structures for atomistic molecular modeling and simulations. *Nucleic Acids Res.* 40:W537–W541.

47. Gomes, T. C. F., and M. S. Skaf. 2012. Cellulose-builder: A toolkit for building crystalline structures of cellulose. *J. Comput. Chem.* 33:1338–1346.

48. Maier, J. A., C. Martinez, …, C. Simmerling. 2015. ff14SB: Improving the Accuracy of Protein Side Chain and Backbone Parameters from ff99SB. *J. Chem. Theor. Comput.* 11:3696–3713.

49. Kirschner, K. N., A. B. Yongye, …, R. J. Woods. 2008. GLYCAM06: A generalizable biomolecular force field. carbohydrates. *J. Comput. Chem.* 29:622–655.

50. Joung, I. S., and T. E. Cheatham. 2008. Determination of alkali and halide monovalent ion parameters for use in explicitly solvated biomolecular simulations. *J. Phys. Chem. B.* 112:9020–9041.

51. Neese, F. 2012. The ORCA program system. *WIREs Comput. Mol. Sci.* 2:73–78.

52. Stephens, P. J., F. J. Devlin, …, M. J. Frisch. 1994. Ab Initio calculation of vibrational absorption and circular dichroism spectra using density functional force fields. *J. Phys. Chem.* 98:11623–11627.

53. Weigend, F., and R. Ahlrichs. 2005. Balanced basis sets of split valence, triple zeta valence and quadruple zeta valence quality for H to Rn: Design and assessment of accuracy. *Phys. Chem. Chem. Phys.* 7:3297–3305.

54. Seminario, J. M. 1996. Calculation of intramolecular force fields from second-derivative tensors. *Int. J. Quant. Chem.* 60:1271–1277.

55. Darden, T., D. York, and L. Pedersen. 1993. Particle mesh Ewald: An N •log(N) method for Ewald sums in large systems. *J. Chem. Phys.* 98:10089–10092.

56. Ryckaert, J. P., G. Ciccotti, and H. J. Berendsen. 1977. Numerical integration of the cartesian equations of motion of a system with constraints: molecular dynamics of n-alkanes. *J. Comput. Phys.* 23:327–341.

57. Salomon-Ferrer, R., A. W. Götz, …, R. C. Walker. 2013. Routine microsecond molecular dynamics simulations with AMBER on GPUs. 2. Explicit solvent particle mesh ewald. *J. Chem. Theor. Comput.* 9:3878–3888.

58. Roe, D. R., and T. E. Cheatham. 2013. PTRAJ and CPPTRAJ: Software for processing and analysis of molecular dynamics trajectory data. *J. Chem. Theor. Comput.* 9:3084–3095.

59. Bissaro, B., B. Streit, …, Å. K. Røhr. 2019. Molecular mechanism of the chitinolytic monocopper peroxygenase reaction. Preprint at bioRxiv. https://doi.org/10.1101/541292.

60. Miao, Y., V. A. Feher, and J. A. McCammon. 2015. Gaussian Accelerated Molecular Dynamics: Unconstrained Enhanced Sampling and Free Energy Calculation. *J. Chem. Theor. Comput.* 11:3584–3595.

61. Miao, Y., W. Sinko, …, J. A. McCammon. 2014. Improved Reweighting of Accelerated Molecular Dynamics Simulations for Free Energy Calculation. *J. Chem. Theor. Comput.* 10:2677–2689.

62. Miao, Y., A. Bhattarai, and J. Wang. 2020. Ligand Gaussian Accelerated Molecular Dynamics (LiGaMD): Characterization of Ligand Binding Thermodynamics and Kinetics. *J. Chem. Theor. Comput.* 16:5526–5547.

63. Swails, J. M., D. M. York, and A. E. Roitberg. 2014. Constant pH replica exchange molecular dynamics in explicit solvent using discrete protonation states: Implementation, testing, and validation. *J. Chem. Theor. Comput.* 10:1341–1352.

64. Becke, A. D. 1988. Density-functional exchange-energy approximation with correct asymptotic behavior. *Phys. Rev. Appl.* 38:3098–3100.

65. Perdew, J. P. 1986. Density-functional approximation for the correlation energy of the inhomogeneous electron gas. *Phys. Rev. B.* 33:8822–8824.

66. Grimme, S., S. Ehrlich, and L. Goerigk. 2011. Effect of the damping function in dispersion corrected density functional theory. *J. Comput. Chem.* 32:1456–1465.

67. Arthur, E. J., and C. L. Brooks, III. 2016. Efficient implementation of constant pH molecular dynamics on modern graphics processors. *J. Comput. Chem.* 37:2171–2180.

68. Kim, S., J. Ståhlberg, …, G. T. Beckham. 2014. Quantum mechanical calculations suggest that lytic polysaccharide monooxygenases use a copper-oxyl, oxygen-rebound mechanism. *Proc. Natl. Acad. Sci. USA.* 111:149–154.

69. Bertini, L., R. Breglia, …, M. Bruschi. 2018. Catalytic Mechanism of Fungal Lytic Polysaccharide Monooxygenases Investigated by First-Principles Calculations. *Inorg. Chem.* 57:86–97.

70. Riley, R., A. A. Salamov, …, I. V. Grigoriev. 2014. Extensive sampling of basidiomycete genomes demonstrates inadequacy of the white-rot/brown-rot paradigm for wood decay fungi. *Proc. Natl. Acad. Sci. USA.* 111:9923–9928.

71. Bissaro, B., A. Várnai, …, V. G. H. Eijsink. 2018. Oxidoreductases and Reactive Oxygen Species in Conversion of Lignocellulosic Biomass. *Microbiol. Mol. Biol. Rev.* 82, e00029-18.

72. Choon, K. K., and E. Roffael. 1990. The Acidity of Five Hardwood Species. *Holzforschung.* 44:53–58.

73. Purahong, W., T. Wubet, …, F. Buscot. 2018. Determinants of deadwood-inhabiting fungal communities in temperate forests: Molecular evidence from a large scale deadwood decomposition experiment. *Front. Microbiol.* 9:2120.

74. Payne, C. M., B. C. Knott, …, G. T. Beckham. 2015. Fungal cellulases. *Chem. Rev.* 115:1308–1448.

75. Kjaergaard, C. H., M. F. Qayyum, …, E. I. Solomon. 2014. Spectroscopic and computational insight into the activation of O2 by the mononuclear Cu center in polysaccharide monooxygenases. *Proc. Natl. Acad. Sci. USA.* 111:8797–8802.

76. Bhadra, M., J. Y. C. Lee, …, K. D. Karlin. 2018. Intramolecular Hydrogen Bonding Enhances Stability and Reactivity of Mononuclear Cupric Superoxide Complexes. *J. Am. Chem. Soc.* 140:9042–9045.

77. Quist, D. A., M. A. Ehudin, …, K. D. Karlin. 2019. Ligand Identity-Induced Generation of Enhanced Oxidative Hydrogen Atom Transfer Reactivity for a Cu II 2 (O 2 •−) Complex Driven by Formation of a Cu II 2 (− OOH) Compound with a Strong O–H Bond. *J. Am. Chem. Soc.* 141:12682–12696.

Biophysical Journal, Volume *123*

Supplemental information

The rotamer of the second-sphere histidine in AA9 lytic polysaccharide monooxygenase is pH dependent

Ingvild Isaksen, Suvamay Jana, Christina M. Payne, Bastien Bissaro, and Åsmund K. Røhr

This file includes:

1. List of Supplementary Figures and Tables
2. Supplementary Figures S1 to S10 and Supplementary Tables 1 to 2
3. Example ORCA input file (stacking His in HIE state and Cu(II))

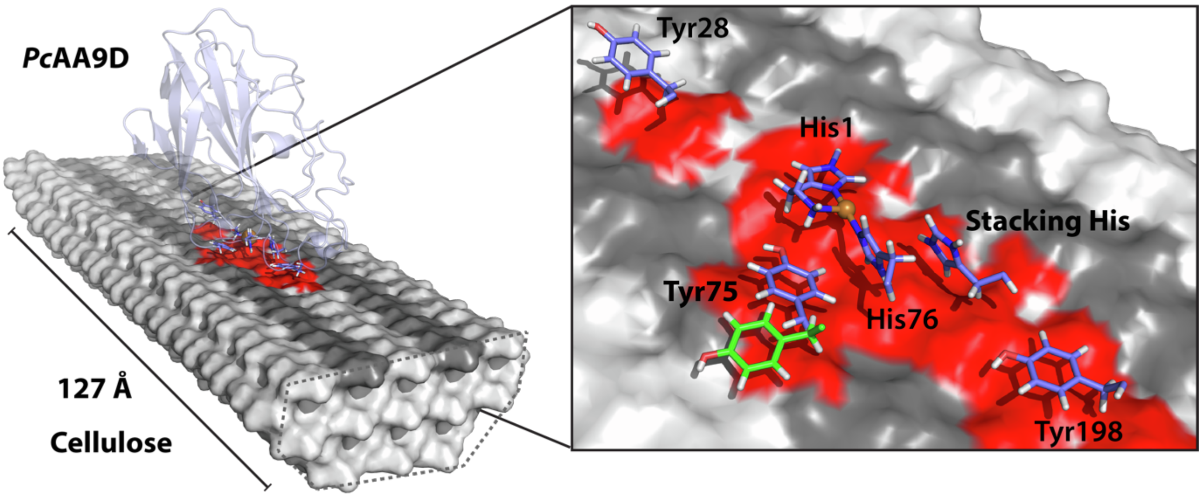

Fig. S1. Constant pH simulations of *Pc*AA9D on cellulose. The direction of cellulose chains forming the crystal is indicated by two chains with dark shades on the surface. The surface interacting with Tyr residues and the active site is colored red. Tyr28 and Tyr198 align along a cellulose chain. The alternative Tyr75 rotamer is indicated in green. The copper atom is shown as orange sphere. When bound to the substrate, the stacking His does not form any hydrogen bonds to the cellulose surface, and its flexibility is sterically restricted by the association of the enzyme with cellulose.

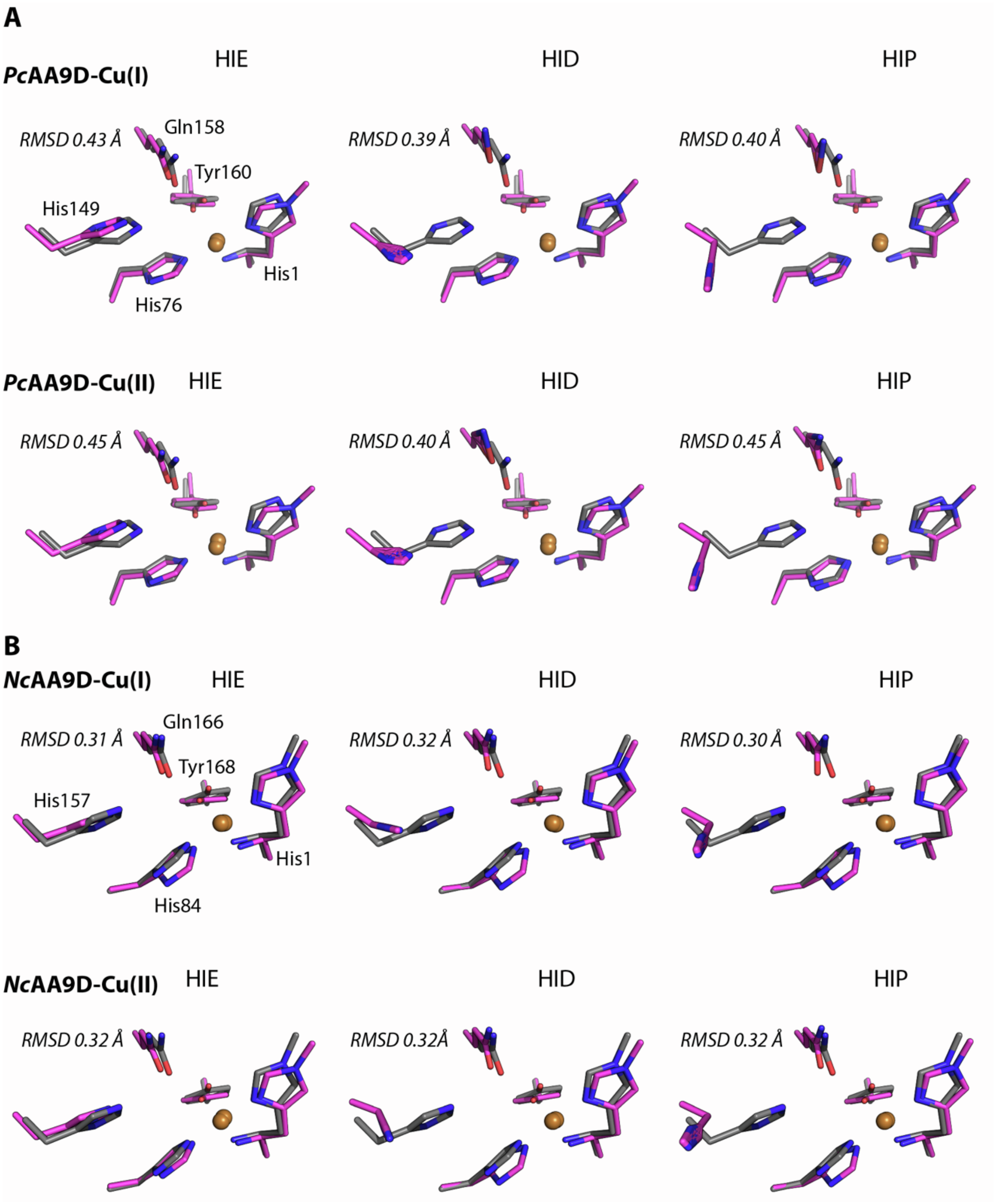

Fig. S2. Comparing averaged structures of *Pc*AA9D and *Nc*AA9D to respective crystal structures. Here, we assess the quality of the AA9 force field parameters developed in this study and evaluate how different protonation states of the stacking His behave during MD simulations in aqueous environments in the absence of substrate. The respective crystal structures, PDBid 4B5Q for *Pc*AA9D and PDBid 4EIR for *Nc*AA9D, are shown with grey carbon, while the averaged MD models from 100 ns of simulation are shown with magenta carbon atoms. The data for *Pc*AA9D are presented in panel A and the *Nc*AA9D data in panel B. The side chain of the HID or HIP state of the stacking His is typically disordered, and this is reflected by the non-physical averaged positions of the atoms in these models. Note that the side chain of the HIP state always has a conformation pointing away from the active site pocket. The RMSD values are calculated using the "super" function in PyMOL and include all the atoms of the displayed amino acids except those of the stacking His.

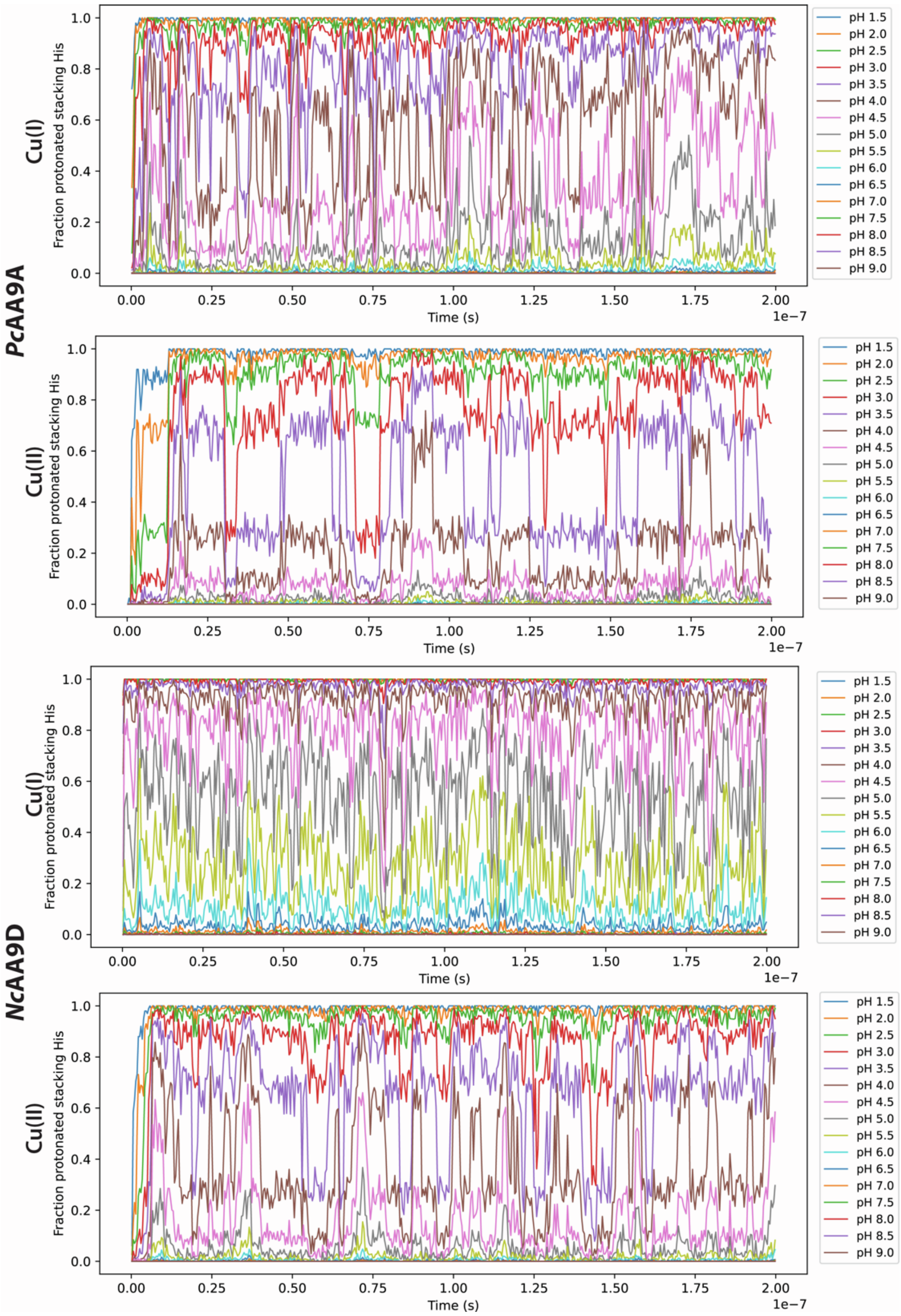

Fig. S3. Fraction of the HIP protonation state throughout the constant pH simulations. The panels show how the fraction of the HIP state changes over time at different pH-values. The data were prepared by sorting the replica exchange cpout files according to pH by the program cphstats (AmberTools23) and then the fractions of HIP were calculated in chunks of 200 000 steps. The simulation time for each pH-replica was 200 ns.

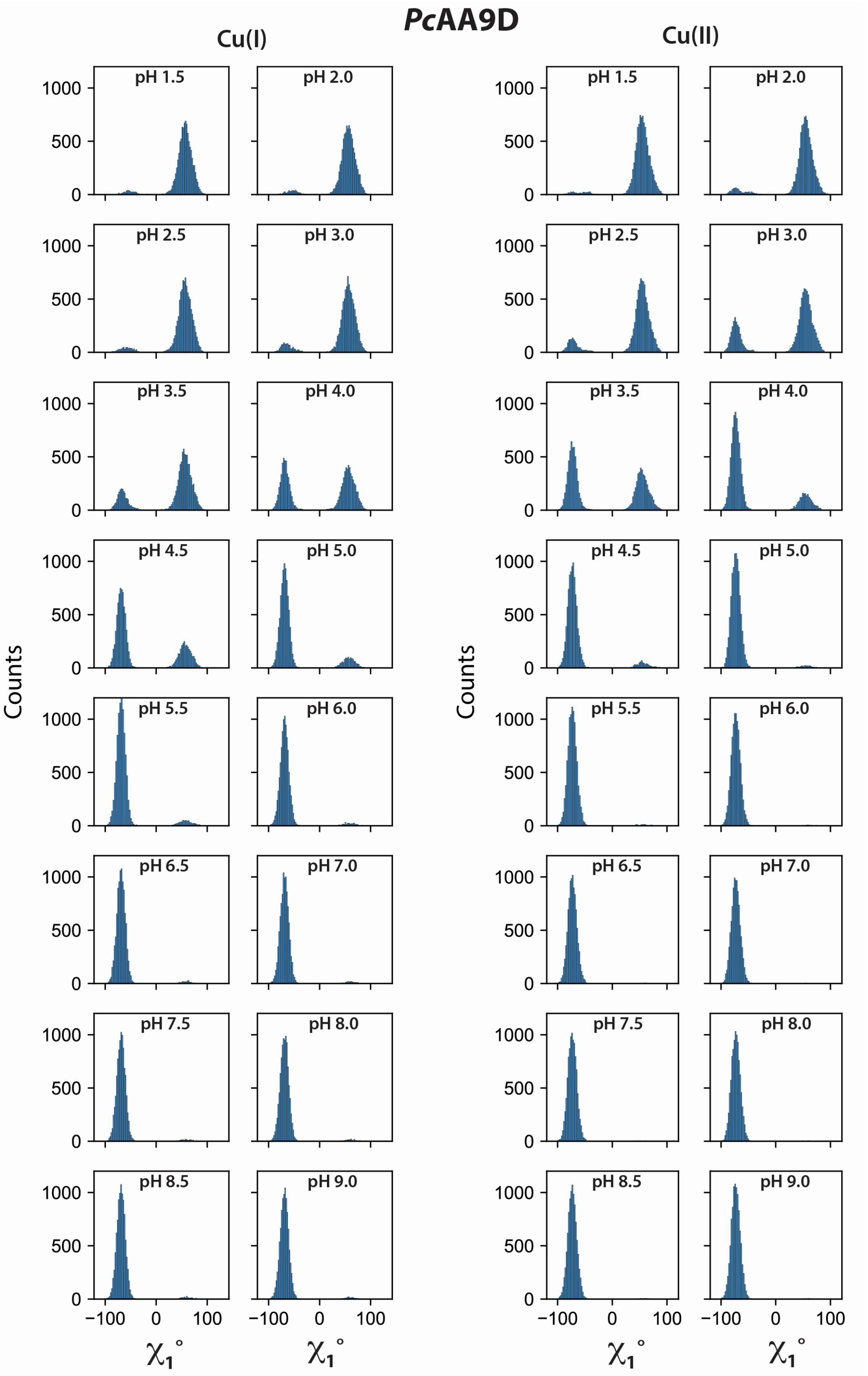

Fig. S4. Each panel show the populations of the stacking His rotamers different pH values in constant pH simulations for *Pc*AA9D. The panels show that two populations of the stacking His rotamers are observed, the "inward" (χ_1 ~ -70°) and "outward" (χ_1 ~ 60°) conformations, at different pH-values. The data were prepared by sorting the replica exchange trajectories according to pH by the program ccptraj (AmberTools23). The simulation time for each pH-replica was 200 ns.

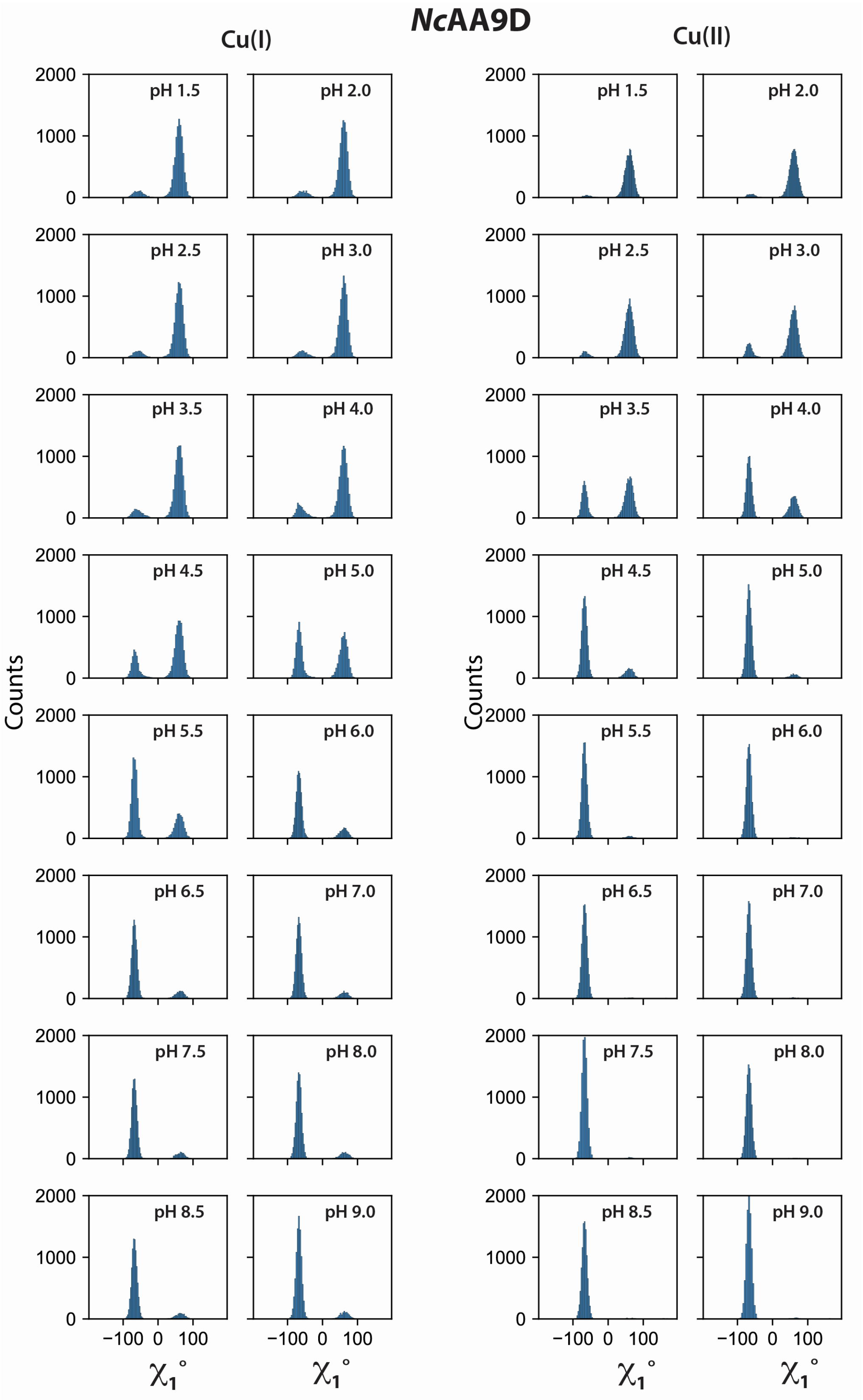

Fig. S5. Each panel show the populations of the stacking His rotamers different pH values in constant pH simulations for *Nc*AA9D. The panels show that two populations of the stacking His rotamers are observed, the "inward" (χ_1 ~ -70°) and "outward" (χ_1 ~ 60°) conformations, at different pH-values. The data were prepared by sorting the replica exchange trajectories according to pH by the program ccptraj (AmberTools23). The simulation time for each pH-replica was 200 ns.

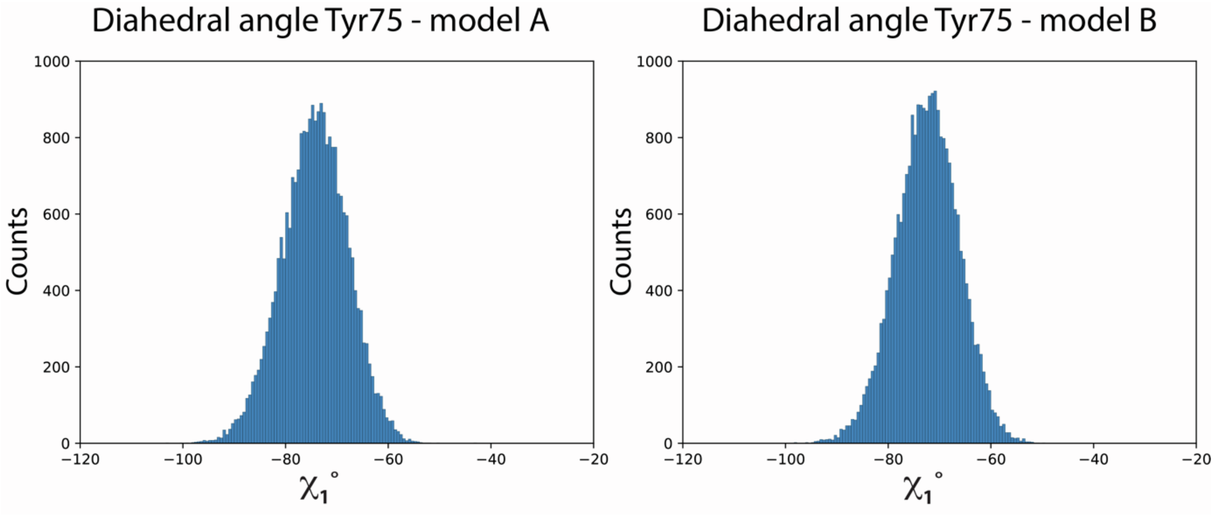

Fig. S6. Monitoring the Tyr75 χ_1 -rotamer in simulations of *Pc*AA9D on cellulose. The figures show how the Tyr160 side chain in the two models A and B, that had the starting dihedrals χ_1 = -64.7° and χ_1 = 160.5°, respectively, display a single population. The distribution of χ_1 falls around -75° for both models in the 500 ns simulations which is the rotamer pointing towards the copper.

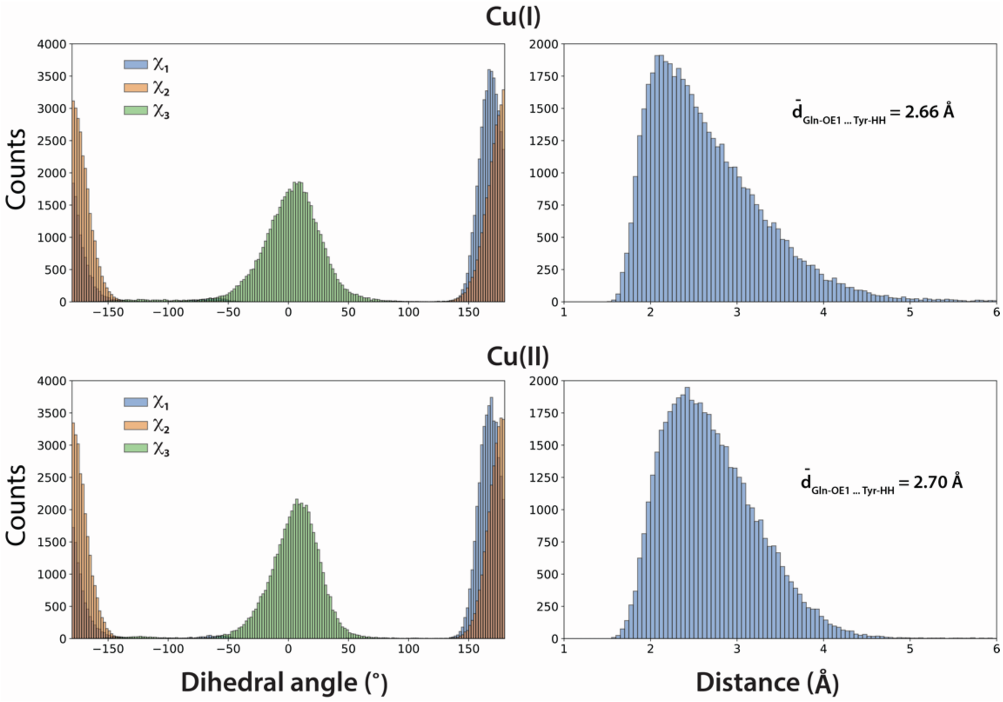

Fig. S7. Monitoring the Gln158 side chain rotamer and distance of Tyr160 interaction in MD simulations of *Pc*AA9D. The figure shows side chain dihedrals χ_1 to χ_3 (standard notation) that were monitored through 100 ns simulations of *Pc*AA9D in the Cu(I) and Cu(II) states. The distributions center around the values measured in the crystal structure (PDBid 4B5A chain A) that are χ_1 = 173.9°, χ_2 = 174.4° and χ_3 = 2.9°. The distance between the

hydrogen of the Tyr160 hydroxyl group and the Gln158 side chain carbonyl has a maximum around 2.3 Å, indicating frequent formation of a hydrogen bond.

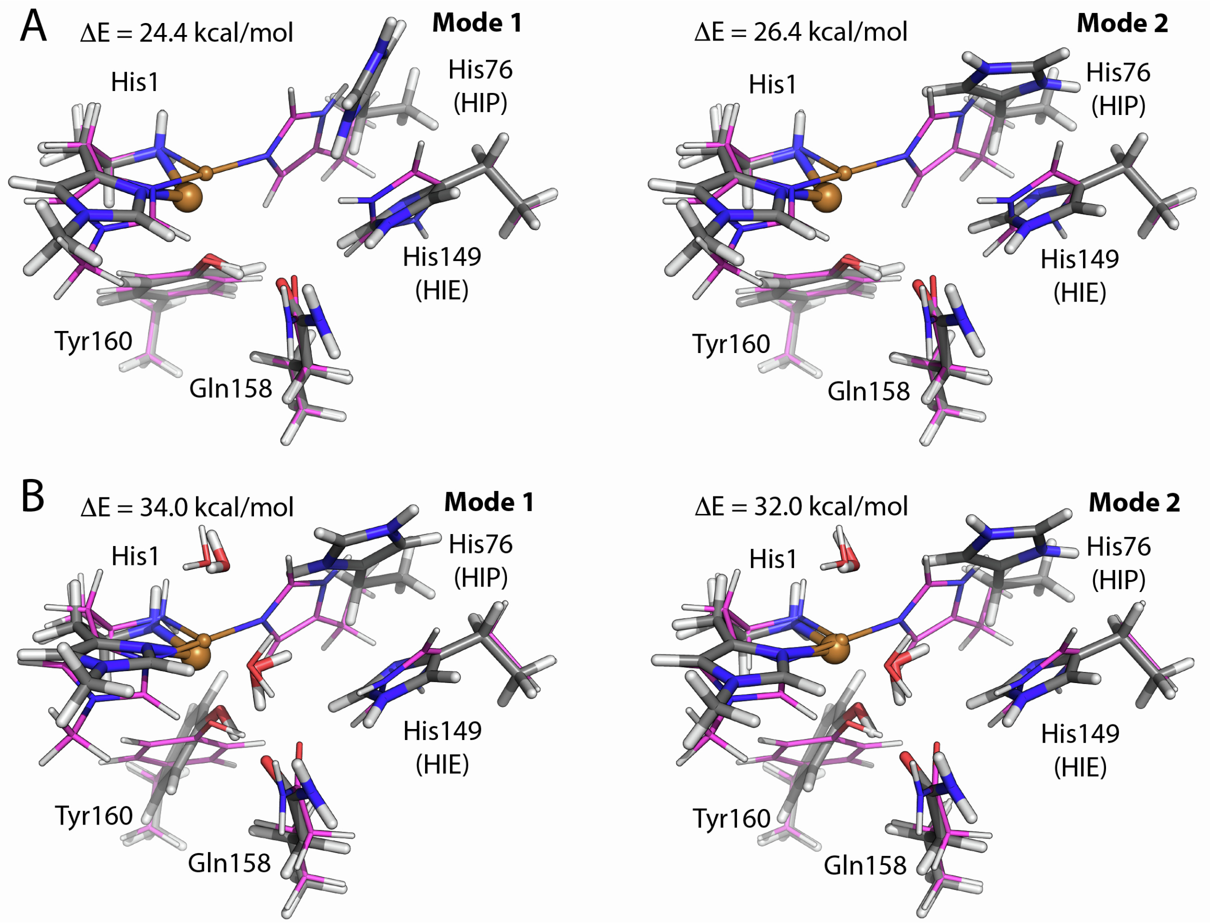

Fig. S8. Geometry optimized active site models of *Pc*AA9D with displaced His76. The DFT geometry optimized models where the models with displaced His76 are compared to the copper coordinating model (with the stacking His in the HIE state) in the Cu(I) state is shown in panel (A). The same comparison with Cu(II) is provided in panel (B). The difference in energy between the copper binding (with the stacking His in the HIP state) and displaced models are indicated, and for all models the copper coordinating version is predicted to be most stable.

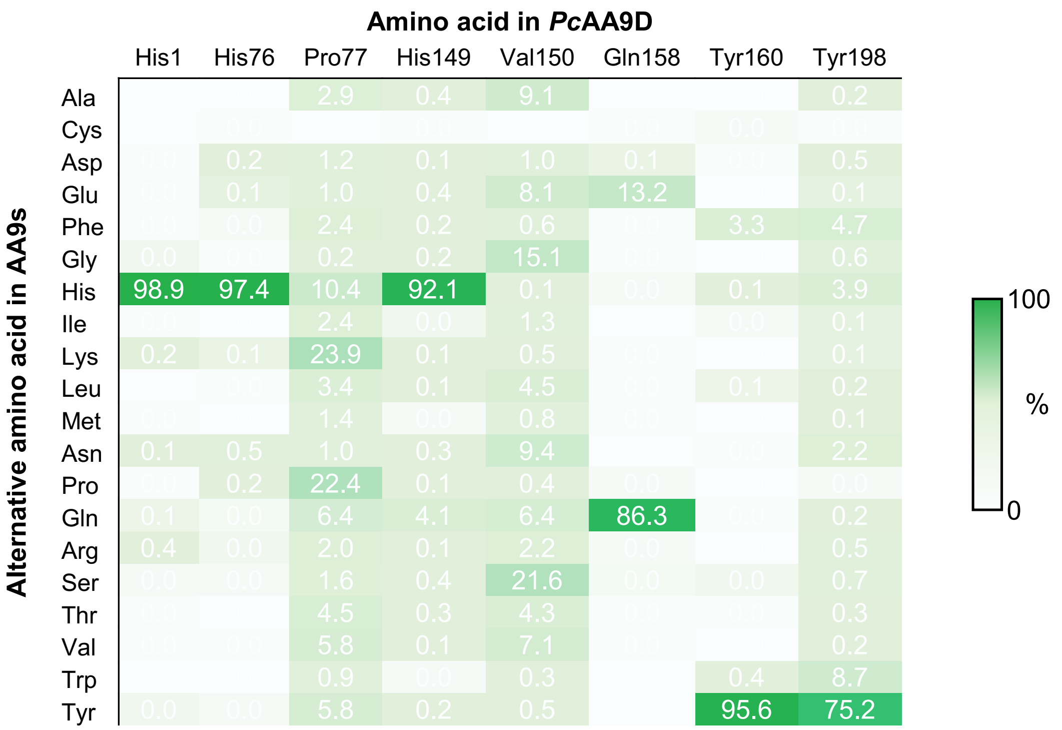

Fig. S9. Heatmap of the conservation score of key active site residues in AA9 LPMOs. The figure shows the conservation score (expressed as percentage), calculated on the basis of a multiple sequence alignment using 16,248 AA9 sequences, for positions equivalent to the copper-coordinating histidines (His1 and His76, *Pc*AA9D numbering), the stacking histidine (His149), catalytic helpers (Gln158 and Tyr160), and other second sphere residues highlighted in the present manuscript (Pro77, Val150 and Tyr198).

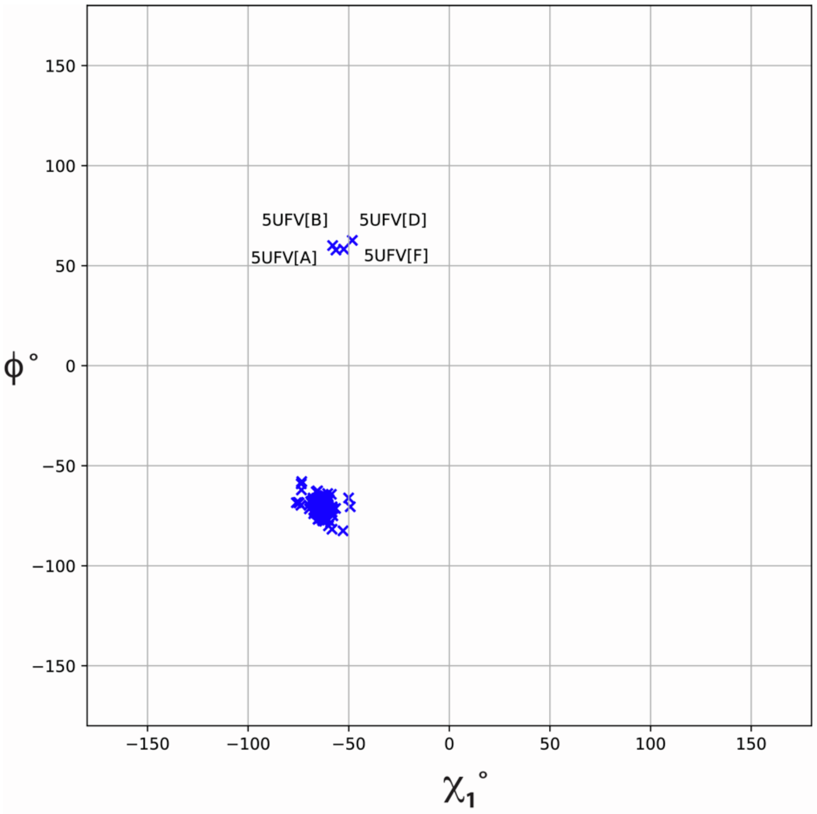

Fig. S10. Dihedral angles of the stacking His in AA9 LPMOs. The figure shows dihedral angles of the backbone N-C_α (ϕ) and the C_α - C_β (χ_1) (see **Fig. 1** for details). There are 89 structures included, some of them have multiple chains. There is a tight correlation between the ϕ and χ_1 values, indicating the a steric clash (see **Fig. 5**) will occur between the proton on the N_δ of the stacking His and the same residue amide hydrogen in most of the structures (the exception is from PDBid 5UFV that display the “outward” conformation in four of the monomers in the asymmetric unit). The included 89 PDB structures are 5NLT, 6YDC, 6YDD, 6YDE, 6YDF, 6H1Z, 6HA5, 6HAQ, 7EXK, 5NNS, 5ACF, 5ACG, 5ACH, 5ACI, 5ACJ, 5N04, 5N05, 5NKW, 5NLN, 5NLO, 5NLP, 5NLQ, 5NLR, 5NLS, 6YDG, 7NIM, 7NIN, 7PQR, 7PXI, 7PXJ, 7PXK, 7PXL, 7PXM, 7PXN, 7PXR, 7PXS, 7PXT, 7PXU, 7PXV, 7PXW, 7PYD, 7PYE, 7PYF, 7PYG, 7PYH, 7PYI, 7PYL, 7PYM, 7PYN, 7PYO, 7PYP, 7PYQ, 7PYU, 7PYW, 7PYX, 7PYY, 7PYZ, 7PZ0, 7NTL, 4EIR, 5TKF, 5TKG, 5TKH, 5TKI, 7T5C, 7T5D, 7T5E, 4QI8, 4EIS, 4D7U, 4D7V, 5FOH, 4B5Q, 7A8V, 2YET, 3ZUD, 7PZ3, 7PZ4, 7PZ5, 7PZ6, 7PZ7, 7PZ8, 7Q1K, 5UFV, 3EII, 3EJA, 2VTC, 5O2W and 5O2X.

Table S1. AMBER force field parameters for the AA9 LPMO active site with Cu(I) or Cu(II)

Bond stretching

	r_0 (Å)	k_r (kcal mol^{-1} Å^{-2})
NB-CU	1.991	83.1
N3-CU	2.090	39.2
OH-CU	2.653$^{Cu(I)}$, 2.371$^{Cu(II)}$	30.0
Extra parameters for methyl-group on His1		
CR-N*	1.376	431.6
CW-N*	1.376	431.6
CT-N*	1.456	334.7

Bond angle bending

	θ_0 (°)	k_θ (kcal mol^{-1} rad^{-2})
CU-N3-CX	117.1	84.4
CU-N3-H	107.0	46.5
N3-CU-NB	92.5	74.1
N3-CU-OH	92.6	37.6
NB-CU-OH	98.0	12.4
NB-CU-NB	161.8	42.2
CU-OH-C	131.1	36.4
CU-OH-HO	109.8	25.0
CU-NB-CR	127.1	77.8
CU-NB-CC	125.5	83.3
CU-NB-CV	125.5	83.3
Extra parameters for methyl-group on His1		
CC-CW-N*	121.6	67.5
N*-CR-NB	125.5	69.4
CR-N*-CW	104.3	70.5
CR-N*-CT	109.5	67.6
H5-CR-N*	123.7	49.2
H4-CW-N*	120.0	50.0
HC-CT-N*	109.4	49.8
CT-N*-CW	109.5	67.6

Charges Cu(I) AA9 active site

Cu(I) 0.1000

N-terminal His		Internal His		Buried Tyr	
Atom name	*Charge*	*Atom name*	*Charge*	*Atom name*	*Charge*
N	-0.4490	CB	-0.0819	CB	0.2619
H1	0.2268	HB2	0.0801	HB2	-0.0322
H2	0.2268	HB3	0.0801	HB3	-0.0322
CA	0.3760	CG	0.0130	CG	-0.1258
HA	0.0132	ND1	-0.1252	CD1	-0.1067
CB	-0.3619	HD1	0.3153	HD1	0.1349
HB2	0.1172	CE1	-0.1127	CE1	-0.2657
HB3	0.1172	HE1	0.1739	HE1	0.1199
CG	0.3015	NE2	-0.0931	CZ	0.3706
ND1	-0.2969	CD2	-0.1080	OH	-0.4024
CE1	-0.1360	HD2	0.1126	HH	0.2803
HE1	0.1879	CA	0.0188		
NEM	0.3217				
CD2	-0.4212				
HD2	0.2180				
CME	-0.3185				
HM1	0.1348				
HM2	0.1348				
HM3	0.1348				
C	0.6123				

Charges Cu(II) AA9 active site

Cu(II)			0.5843		
N-terminal His		Internal His		Buried Tyr	
Atom name	*Charge*	*Atom name*	*Charge*	*Atom name*	*Charge*
N	-0.5957	CB	-0.1166	CB	0.2510
H1	0.3166	HB2	0.1063	HB2	-0.0183
H2	0.3166	HB3	0.1063	HB3	-0.0183
CA	0.2432	CG	0.1282	CG	-0.0978
HA	0.0765	ND1	-0.2043	CD1	-0.1255
CB	-0.1877	HD1	0.3551	HD1	0.1526
HB2	0.1129	CE1	-0.0776	CE1	-0.1884
HB3	0.1129	HE1	0.1947	HE1	0.1111
CG	0.0810	NE2	-0.1099	CZ	0.2680
ND1	-0.1266	CD2	-0.1978	OH	-0.4361
CE1	-0.2910	HD2	0.1114	HH	0.2875
HE1	0.2218	CA	0.25		
NEM	-0.1057				
CD2	0.1865				
HD2	0.2375				
CME	-0.2949				
HM1	0.1481				
HM2	0.1481				
HM3	0.1481				
C	0.6518				

Table S2. Analysis of populated states for His149 in cellulose-bound *Pc*AA9D in constant pH-replica exchange molecular dynamics simulations (starting model A and B)

Solvent pH	Fraction of protonated states recorded in 200 ns simulations for each pH					
	State 0 (HIP)		*State 1 (HID)*		*State 2 (HIE)*	
	**Model A**	**Model B**	**Model A**	**Model B**	**Model A**	**Model B**
1.50	0.000000	0.000002	0.004017	0.006266	0.995983	0.993732
2.00	0.000000	0.000001	0.004111	0.006476	0.995889	0.993523
2.50	0.000000	0.000000	0.004108	0.006498	0.995892	0.993502
3.00	0.000000	0.000000	0.004177	0.006443	0.995823	0.993557
3.50	0.000000	0.000000	0.004234	0.006401	0.995766	0.993599
4.00	0.000000	0.000000	0.004144	0.006296	0.995856	0.993704
4.50	0.000000	0.000000	0.004225	0.006193	0.995775	0.993807
5.00	0.000000	0.000000	0.004179	0.006039	0.995821	0.993961
5.50	0.000000	0.000000	0.004157	0.006173	0.995843	0.993827
6.00	0.000000	0.000000	0.004130	0.006208	0.995870	0.993792
6.50	0.000000	0.000000	0.004148	0.006185	0.995852	0.993815
7.00	0.000000	0.000000	0.004096	0.006282	0.995904	0.993718
7.50	0.000000	0.000000	0.004101	0.006236	0.995899	0.993764
8.00	0.000000	0.000000	0.004062	0.006245	0.995938	0.993755
8.50	0.000000	0.000000	0.004092	0.006187	0.995908	0.993813
9.00	0.000000	0.000000	0.004132	0.006191	0.995868	0.993809

Example ORCA input file:

```
# Orca input file
! UKS B3LYP OPT def2-SVP D4 TightSCF SlowConv

%base "b3lyp_opt"

%pal nprocs 10
    end

%basis
  newGTO Cu "def2-TZVPP" end
end

%geom
   ConnectFragments

   {1 2 C 15 23 }
   {1 3 C 15 38 }
   {1 4 C 15 53 }
   {1 5 C 15 68 }
   {1 6 O 15 87 }
   {1 7 O 15 88 }
   {1 8 O 15 91 }
   end
end

%scf
MaxIter 1500
DIISMaxEq 15
end

* xyz 2 2
N(1)   3.36069110761946     6.25010312328851    -15.35543576294581
H(1)   2.90695213375984     7.15845360886818    -15.52680163412305
H(1)   2.94976768176188     5.88514357866140    -14.48290882281649
C(1)   4.82849355357674     6.44022855073708    -15.15029603224286
H(1)   5.19747371120119     5.44630273419179    -14.82137099493967
C(1)   5.50741521182581     6.78457414659860    -16.47967652403444
H(1)   4.97419534099623     7.62376672611532    -16.97290914018203
H(1)   6.52564175887847     7.16267699992295    -16.26399994621672
C(1)   5.65000430013041     5.60273431070813    -17.41244343800138
N(1)   4.60491859329780     4.83026779422226    -17.77972134502170
C(1)   5.03453185690099     3.86145663412101    -18.61148475461291
H(1)   4.47435444009080     3.06202039279497    -19.09026594951698
N(1)   6.35801018189872     4.01523613272871    -18.77588060280792
```

C(1)	6.76670916585375	5.07994554815823	-18.05384558200822
H(1)	7.81142597506136	5.41108752452430	-18.04705962000941
C(1)	5.13742755943837	7.44157735162310	-14.06587436334866
C(1)	7.22370237407258	3.14590066017460	-19.59512346189016
H(1)	7.70546171456016	3.73800504341613	-20.39616073351435
H(1)	6.60715563207852	2.35106819004931	-20.05033836753547
H(1)	8.00300041461844	2.68594395600216	-18.95855362608271
H(1)	6.22696975932433	7.48046045340256	-13.86678321344335
H(1)	4.81449833151011	8.46769306692085	-14.34446776348778
H(1)	4.64035063753438	7.17253990549299	-13.11202913578676
C(2)	-3.09350422707110	5.23310231331244	-14.24881716939278
C(2)	-2.02911519606364	4.17167051820058	-14.49496454729120
H(2)	-1.52548180471928	3.89397808741075	-13.54764643633108
H(2)	-2.48674431181304	3.23152481892388	-14.88328856903453
C(2)	-0.95081193693749	4.57322893910912	-15.47317409418563
N(2)	-1.21992477688773	5.09578258990847	-16.69117941411969
H(2)	-2.15897190279197	5.24478994404343	-17.07135042139577
C(2)	-0.06343772013575	5.31305174786574	-17.34448011139885
H(2)	0.01417447601068	5.77224031990922	-18.33559612522308
N(2)	0.94762730585045	4.93855789374098	-16.54004250117959
C(2)	0.43491877511506	4.47123269224226	-15.38617893778095
H(2)	1.04535496393122	4.06374292712683	-14.57337208708744
H(2)	-3.83421043013390	4.86903076893024	-13.51080245371701
H(2)	-2.65897259452025	6.17362345189065	-13.85394096854520
H(2)	-3.66623885372595	5.48120609742847	-15.16754577947917
C(3)	-4.87822942918604	0.33248180216325	-18.03263060511988
C(3)	-4.36349689624197	1.75993321159828	-18.17094871265023
H(3)	-4.72866103437339	2.21833947770702	-19.11380524339295
H(3)	-4.77531327847072	2.38078072576520	-17.34447207025398
C(3)	-2.85545557324195	1.87711884689340	-18.12822809240209
N(3)	-2.13190376850070	1.51407306426481	-17.05226415570522
C(3)	-0.82821304784465	1.73836684717977	-17.28922255998767
H(3)	0.02162696114094	1.52769086268678	-16.63208401618738
N(3)	-0.71428585124539	2.26664963847666	-18.52444518560130
H(3)	0.15507300649415	2.40541290189572	-19.03900914015994
C(3)	-1.94713929192272	2.36757808605283	-19.06616367415684
H(3)	-2.09536360327059	2.73346248058655	-20.09007674541300
H(3)	-5.98539672495845	0.31281972865203	-18.00412886328978
H(3)	-4.54878785965205	-0.30170826880435	-18.88062457595024
H(3)	-4.50212273509173	-0.13070976997883	-17.09857702616906
C(4)	2.46342538909779	-2.24862361281552	-14.55483415153107
C(4)	2.43531365089472	-0.96005681587789	-15.36616351719054
H(4)	1.50057273229063	-0.40124586219339	-15.14582407920657
H(4)	3.25900650323302	-0.28333736340959	-15.05605543208095
C(4)	2.51598307042395	-1.18594418817485	-16.86857868823683
H(4)	3.48478675771435	-1.65559655674695	-17.15423103022532
H(4)	1.73444672192463	-1.90676435430981	-17.20088429479965

```
C(4)   2.35083347811388     0.09366153498599    -17.66523205342749
O(4)   2.22486716490969     1.18499829453458    -17.10305739913178
N(4)   2.35926421221942    -0.03434202270625    -18.99211750959856
H(4)   2.20157744164507     0.77081884795728    -19.59809683237365
H(4)   2.42141018006469    -0.94852703665452    -19.44412533749113
H(4)   2.40537736862526    -2.03727102630952    -13.46901328757420
H(4)   1.61069533255103    -2.91160022391984    -14.80995962434700
H(4)   3.39508724889639    -2.82666756925615    -14.72887624710202
C(5)   4.97723995173900     1.26048857221720     -9.48402082763512
C(5)   4.22328631232703     2.53090144084113     -9.86986930770160
H(5)   3.22138840661584     2.54421233232269     -9.39281469842577
H(5)   4.76481846306868     3.42474973098565     -9.49355837190387
C(5)   4.07428790616272     2.62128327160808    -11.37658215289970
C(5)   5.12229082333205     3.08658822698271    -12.16541305129597
H(5)   6.05825145652264     3.41489115357940    -11.68541441478491
C(5)   5.01002258852123     3.14509804719640    -13.54926797190256
H(5)   5.86601323369725     3.47953172816064    -14.16085320408370
C(5)   3.83975649485525     2.72039503920325    -14.15643320381172
O(5)   3.72647065078571     2.77485847197022    -15.53331738716652
H(5)    3.091854 2.135455 -15.884828
C(5)   2.78879873205647     2.24628340734727    -13.39566849630879
H(5)   1.88615269643895     1.87467668568720    -13.90289506449421
C(5)   2.91306794327773     2.18876125062277    -12.01178361543141
H(5)   2.07688319656763     1.79725463304458    -11.41140395926209
H(5)   5.99121299613359     1.24047652088591     -9.93362342786603
H(5)   5.09697279339550     1.17748332700502     -8.38491523963758
H(5)   4.44199387016382     0.35410796277802     -9.83411442025919
Cu(6)  2.83158546196694     4.99679449457430    -17.00300911912393
O(7)   2.63128933198794     3.92383420771523    -18.45571445751321
H(7)   1.76574533438357     4.07592516012055    -18.89735363986935
H(7)   2.49026671907397     2.99769504217166    -18.04671231529538
O(8)   2.87202350861426     6.71966816188550    -17.86825841557105
H(8)   2.20359498245672     7.43424300621560    -17.94524205831994
H(8)   3.44979415686138     6.84040209192311    -18.65130088032331
*

##############################################################################
######
# JOB 2
##############################################################################
######

$new_job

%base "b3lyp_sp"

! UKS B3LYP def2-TZVPP D4 TightSCF SlowConv
```

```
%pal nprocs 10
    end

%scf
MaxIter 1500
DIISMaxEq 15
end

* xyzfile 2 2
```
